# Mouse Modeling Dissecting Macrophage–Breast Cancer Communication Uncovered Roles of PYK2 in Macrophage Recruitment and Breast Tumorigenesis

**DOI:** 10.1002/advs.202105696

**Published:** 2022-01-29

**Authors:** Anna‐Katharina Müller, Ulrike A. Köhler, Sébastien Trzebanski, Yaron Vinik, Harsha Mohan Raj, Jean‐Antoine Girault, Nir Ben‐Chetrit, Antonio Maraver, Steffen Jung, Sima Lev

**Affiliations:** ^1^ Molecular Cell Biology Department Weizmann Institute of Science Rehovot 76100 Israel; ^2^ Immunology Department Weizmann Institute of Science Rehovot 76100 Israel; ^3^ Institut du Fer à Moulin Inserm and Sorbonne University UMR‐S1270 Paris France; ^4^ Sandra and Edward Meyer Cancer Center Weill Cornell Medicine New York NY 10065 USA; ^5^ Institut de Recherche en Cancérologie de Montpellier Inserm U1194 – Université Montpellier Montpellier 34090 France

**Keywords:** breast cancer, crosstalk signaling, Notch1, PYK2, tumor‐associated macrophages, tumor microenvironment

## Abstract

Macrophage infiltration in mammary tumors is associated with enhanced tumor progression, metastasis, and poor clinical outcome, and considered as target for therapeutic intervention. By using different genetic mouse models, the authors show that ablation of the tyrosine kinase PYK2, either in breast cancer cells, only in the tumor microenvironment, or in both, markedly reduces the number of infiltrating tumor macrophages and concomitantly inhibits tumor angiogenesis and tumor growth. Strikingly, PYK2 ablation only in macrophages is sufficient to induce similar effects. These phenotypic changes are associated with reduced monocyte recruitment and a substantial decrease in tumor‐associated macrophages (TAMs). Mechanistically, the authors show that PYK2 mediates mutual communication between breast cancer cells and macrophages through critical effects on key receptor signaling. Specifically, PYK2 ablation inhibits Notch1 signaling and consequently reduces CCL2 secretion by breast cancer cells, and concurrently reduces the levels of CCR2, CXCR4, IL‐4Rα, and Stat6 activation in macrophages. These bidirectional effects modulate monocyte recruitment, macrophage polarization, and tumor angiogenesis. The expression of PYK2 is correlated with infiltrated macrophages in breast cancer patients, and its effects on macrophage infiltration and pro‐tumorigenic phenotype suggest that PYK2 targeting can be utilized as an effective strategy to modulate TAMs and possibly sensitize breast cancer to immunotherapy.

## Graphical abstract

This study describes different genetic models to dissect macrophage–breast cancer communication and demonstrates the critical role of the non‐receptor tyrosine kinase PYK2 in this mutual process. PYK2 modulates key signaling pathways mediated by CCL2/CCR2, CXCR4, and IL‐4Rα/pSTAT6, and positively regulates macrophage recruitment and polarization, tumor angiogenesis, and tumor growth.

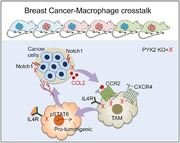

## 1 Introduction

TAMs, the most abundant immune cells in the tumor microenvironments (TME), originate from circulating blood monocytes and tissue resident macrophages.^[^ ^1^, ^2^ ^]^ Monocytes are initially recruited into the TME by multiple chemokines (CCL2, CSF‐1, CXCL12) secreted by malignant and stromal cells, and subsequently differentiate locally into TAMs.^[^ ^2^ ^]^ In breast cancer, and particularly in triple negative breast cancer (TNBC), infiltrated TAMs facilitate tumor growth, enhance angiogenesis, immunosuppression, and drug resistance, and are highly correlated with poor prognosis.^[^ ^3^, ^4^ ^]^ These pro‐tumorigenic effects are thought to be mediated by mutual communication between breast cancer cells, TAMs, and other cells of the TME.^[^ ^5^ ^]^

A wide spectrum of TAMs displaying heterogeneous phenotypes has been identified in the TME.^[^ ^6^ ^]^ Although oversimplified, TAMs have been classified into two extreme phenotypes: classically activated (M1) and alternatively activated (M2) macrophages. Inflammatory anti‐tumorigenic M1‐like macrophages are typically activated by Th1 cytokines, such as IFN‐*γ* and CSF‐2 or by lipopolysaccharide. These cells are characterized by secretion of pro‐inflammatory cytokines, including TNF*‐α*, IFN*‐γ*, IL‐12, and IL‐23,^[^ ^7^ ^]^ by high expression of nitric oxide synthase 2 (NOS2) and production of NO or reactive oxygen species, and by promotion of Th1 lymphocyte development.^[^ ^8^ ^]^ By contrast, immunosuppressive pro‐tumorigenic M2‐like macrophages are activated by Th2 cytokines including IL‐4, IL‐13, and IL‐10,^[^ ^9^ ^]^ which leads to activation of several transcription factors including STAT6, upregulation of certain chemokines (such as CCL17, CCL22, and CCL24) and anti‐inflammatory cytokines (such as IL‐10, TGF*‐β*), which promote the development of Th2 lymphocytes and regulatory T cells (Tregs). M2‐like macrophages express specific cellular markers, including ARG1, YM1, FIZZ1, and the cell–surface scavenger receptors MRC1/CD206 and CD163.^[^ ^6^, ^10^, ^11^ ^]^ These cells support tumor progression and metastasis through several mechanisms, including secretion of numerous growth factors, cytokines, and ECM‐remodeling molecules and metalloproteinases that promote tumor growth, migration, and angiogenesis.^[^ ^12^ ^]^

Despite the characteristic features of M1/M2‐like macrophages, these two extreme activation states display phenotypic plasticity and can switch in response to microenvironmental cues (inflammation, pathogens, tumor cell death) from anti‐inflammatory/pro‐tumorigenic (M2‐like) toward pro‐inflammatory/anti‐tumorigenic (M1‐like), which suppress tumor growth.^[^ ^7^ ^]^ M1‐ and M2‐like TAMs share characteristic features with MHCII^high^ and MHCII^low^ TAMs, respectively. MHCII^high^ TAMs, which usually appear at early stages of tumor development, display tumor‐suppressive activity, while MHCII^low^ TAMs are more dominant at progression phases and exhibit tumor‐promoting effects.^[^ ^13^ ^]^ Increasing evidence suggests that pro‐tumorigenic TAMs are predominant in the TME of most cancers.^[^ ^6^ ^]^ Therefore, targeting their survival/proliferation, recruitment, and/or polarization into an M2‐like pro‐tumorigenic phenotype have been proposed as effective strategies for breast cancer therapy, possibly in combination with other therapeutic agents like chemotherapy or immune checkpoint blockade (ICB).^[^ ^14^, ^15^ ^]^

We previously showed that co‐targeting of the non‐receptor tyrosine kinase PYK2 and EGFR could be beneficial for basal‐like breast cancer with high‐to‐moderate expression levels of EGFR.^[^ ^16^, ^17^ ^]^ In addition, PYK2 expression exhibits significant correlation with breast cancer grade and lymph node metastasis.^[^ ^18^, ^19^ ^]^ PYK2 is also highly expressed in macrophages and affects macrophage infiltration into inflammatory regions, macrophage motility and phagocytosis, as well as macrophage‐mediated inflammatory responses.^[^ ^20^, ^21^, ^22^ ^]^ Previous studies have shown that coinhibition of PYK2 together with its closely related kinase FAK using a dual‐kinase inhibitor reduced TAMs in xenograft models of breast cancer,^[^ ^23^ ^]^ and recent studies suggest that activation of PYK2 by tumor cell‐derived spondin 2 (SPON2), an extracellular matrix glycoprotein that binds and activates integrin *β*1 signaling, is essential for macrophage infiltration in colorectal cancer.^[^ ^24^ ^]^ These studies imply that PYK2 may also play a role in breast cancer‐macrophage communication. However, the influence of PYK2 on TAMs has not been thoroughly defined, and currently, a highly potent and selective PYK2 inhibitor is not available. Furthermore, a systemic administration of pharmacological drug simultaneously affects both the tumor cells and the TME, and thus cannot be used to dissect the discrete contribution of each compartment for tumor development. In this study, we applied genetic approaches to ablate the PYK2 gene (*PTK2B*) either in breast cancer cells using the CRISPR/Cas9 technology, in the TME (total knockout mice), or selectively in macrophages (*Cx3cr1‐Cre^tg/wt^*/*PYK2^f/f^* mice). These genetic models enabled us to selectively dissect the functional impact of PYK2 in macrophages, tumor cells, and in macrophage–tumor cell communication, and to uncover key pathways that are regulated by PYK2 and modulate TAM recruitment and polarization.

## 2 Results

### 2.1 PYK2 Modulates TAM Infiltration and Breast Cancer Growth

**Figure 1:**
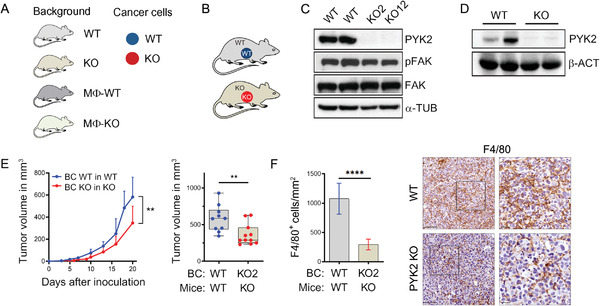
Ablation of PYK2 reduced breast cancer growth and TAM numbers. A) Schematic overview of the different mouse backgrounds and breast cancer cell genotypes used throughout the manuscript figures. Mouse background: gray (WT), gold (total PYK2 KO), dark gray (MΦ‐WT), and light green (MΦ‐KO). Breast cancer cells: WT (blue) or PYK2 KO (red) EO771 cells. B) Schematic presentation of the analyzed tumor models: wild‐type (WT) or PYK2 KO (KO) EO771 cells were orthotopically injected into WT or PYK2 KO (KO) mice. C,D) Western blot analyses confirmed PYK2 deletion in the two CRISPR/Cas9‐mediated PYK2 KO EO771 cell lines (KO2 and KO12) (C), and in normal breast tissue from PYK2 KO mice (D). E) Tumor growth curves over 20 days show the mean tumor volume at the indicated time points following EO771 implantation (WT and KO) in WT mice (*n* = 10) or PYK2 KO mice (*n* = 11); statistical significance was determined by mixed‐effects model. Volume of individual tumor at day 20 is shown in the box plot; statistical significance was determined by Welch *t*‐test. F) Quantification of F4/80‐positive cells in tumor sections from WT (*n* = 9) and PYK2 KO (*n* = 6) mice at day 20 following implantation (Experimental Section). Statistical significance was determined by Welch *t*‐test. Representative immunohistochemical images are shown. Scale bar: 50 µm. BC (breast cancer).

To define the influence of PYK2 on breast cancer growth, we established a syngeneic breast cancer model by orthotopic implantation of wild‐type (WT) and PYK2 knockout (KO) breast cancer (BC) cells into WT or PYK2^ko/ko^ (PYK2 KO) C57BL/6 mice (**Figure** **1**A,B),^[^ ^25^, ^26^ ^]^ respectively. In the absence of a selective PYK2 inhibitor, these mouse models recapitulate systemic drug administration in an immunocompetent microenvironment. The breast cancer cell line EO771, derived from a spontaneous mammary tumor in C57BL/6 mice,^[^ ^27^, ^28^ ^]^ was used to establish PYK2 KO cell lines by CRISPR/Cas9 technology (Experimental Section). Several EO771 clones were generated (Figure S1A, Supporting Information) and two independent clones (KO2 and KO12) of two distinct sgRNAs were selected for further analysis (Figure 1C). Control (WT) and PYK2 KO EO771 cells (KO2) were implanted into the mammary fat pad of WT and PYK2 KO mice. The absence of PYK2 in breast tissue of PYK2 KO mice was confirmed by Western blotting (Figure 1D).

Tumor growth was measured over time with calipers until reaching a volume of ≈0.6 cm^3^ (Figure 1E). As shown in Figure 1E, implantation of PYK2 KO BC cells into the mammary fat pad of PYK2 KO mice significantly attenuated tumor growth compared to WT control, and significantly reduced tumor volume (by ≈40%, *p* = 0.004) at the endpoint (day 20). Hematoxylin and Eosin (H&E) staining of tumor sections (Figure S1B, Supporting Information) revealed a less condensed tumor tissue in the PYK2 KO compared to WT control, and immunohistochemistry (IHC) analysis for PYK2 confirmed the ablated genotype in the tumor sections (Figure S1C, Supporting Information). Importantly, despite the reduced tumor growth of implanted PYK2 KO EO771 cells (KO2, KO12), these cells had the same proliferation rate as WT cells in vitro (Figure S1D, Supporting Information), implying that the TME of PYK2 KO mice imposes tumor inhibitory effects.

It is well known that PYK2 is expressed in different cell types of the TME,^[^ ^29^ ^]^ and our immunofluorescence (IF) analysis confirmed its high protein expression in various immune cells (T cells, NK cells, monocytes), moderate expression in endothelial cells and a very low expression in fibroblasts (Figure S1E–I, Supporting Information). Since PYK2 ablation had profound effects on macrophages,^[^ ^20^ ^]^ and TAMs are the major component of the TME that strongly contribute to tumor development and progression,^[^ ^30^ ^]^ we examined the influence of PYK2 KO on macrophage infiltration. We used the murine macrophage marker F4/80 and IHC analysis. As seen in Figure 1F, the number of F4/80‐positive macrophages was substantially reduced in PYK2 KO tumors compared to the WT. These results suggest that the inhibitory effects of PYK2 ablation on tumor growth were mediated, at least in part, by reduced number of TAMs and their pro‐tumorigenic effects.

### 2.2 Dissecting the Distinctive Impact of PYK2 Deficiency in Breast Cancer Cells and the TME

**Figure 2:**
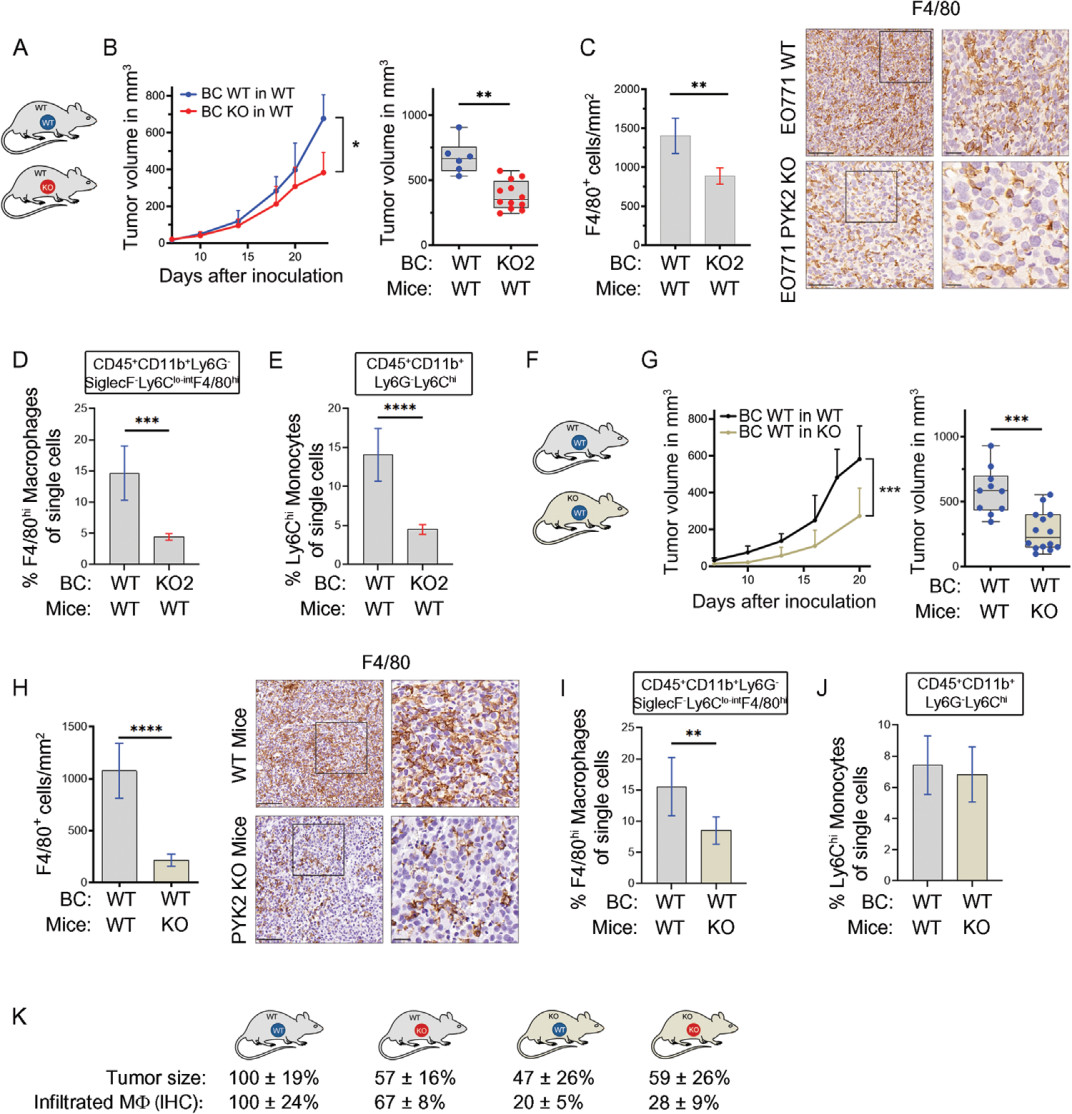
PYK2 affects macrophage infiltration and breast cancer growth in different mouse models. A) Schematic presentation of the analyzed tumor models; wild‐type (WT) or PYK2 KO (KO) EO771 BC cells were orthotopically injected into WT C57B/L6 mice. B) Tumor growth curves over 24 days show the mean tumor volume at the indicated time points following implantation of WT (*n* = 6) and PYK2 KO2 (*n* = 12) EO771 cells into WT mice; statistical significance was determined by 2‐way ANOVA. Volume of individual tumors at day 24 is shown in the box plot; statistical significance was determined by Welch *t*‐test. C) Quantification of F4/80‐stained cells on sections from WT and BC PYK2 KO tumors in WT mice (WT/WT vs KO2/WT). Representative IHC images of WT/WT (*n* = 6) and KO2/WT (*n* = 5) are shown. Scale bar: 100 µm, 25 µm (for inset). Statistical significance was determined by Welch *t*‐test. D,E) Flow cytometry analysis of WT/WT (*n* = 8) and KO2/WT (*n* = 8) tumors depicted as percentages of single cells showing F4/80^hi^ macrophages (CD45^+^CD11b^+^Ly6C^lo‐int^Ly6G^−^SiglecF^−^F4/80^hi^) (D) and Ly6C^hi^ monocytes (CD45^+^CD11b^+^Ly6C^hi^Ly6G^−^SiglecF^−^) (E). Statistical significance was determined by Welch *t*‐test. F) Schematic representation of the analyzed tumor model: WT BC cells (EO771) were injected into WT and PYK2 KO mice. G) Tumor growth curves over 20 days show the mean tumor volume at the indicated time points following implantation of WT EO771 cells into WT (*n* = 10) and PYK2 KO (*n* = 14) mice; statistical significance was determined by mixed‐effects model. Volume of individual tumor at day 20 is shown in the box plot; statistical significance was determined by Welch *t*‐test for individual time points. H) Quantification of F4/80‐stained cells on sections from WT tumors in WT and PYK2 KO mice (WT/WT vs WT/KO). Representative IHC images of WT/WT (*n* = 9, the same as in Figure 1F) and WT/KO (*n* = 6) are shown. Scale bar: 100 µm, 25 µm (for inset). The same WT/WT data was used in panels G, H and in Figures 1E, F. I,J) Flow cytometric analysis of WT/WT (*n* = 8) and WT/KO (*n* = 8) tumors depicted as percentages of single cells showing F4/80^hi^ macrophages (CD45^+^CD11b^+^Ly6C^lo‐int^Ly6G^−^SiglecF^−^F4/80^hi^) (I) and Ly6C^hi^ monocytes (CD45^+^CD11b^+^Ly6C^hi^Ly6G^−^SiglecF^−^) (J). K) Graphical summary of tumor volume and relative numbers of tumor‐associated macrophages (IHC) in the different models studied. Effects were calculated as fold of control (%).

To explore the possibility that PYK2 KO reduces TAM numbers, thereby suppressing their pro‐tumorigenic effects and attenuates BC growth, we systematically dissected the impact of PYK2 ablation either in the BC cells or in the TME using different mouse models. First, we evaluated the impact of PYK2 ablation only in BC cells by comparing tumor growth of WT and PYK2 KO EO771 cells implanted in WT mice (**Figure** **2**A). As shown in Figure 2B and Figure S2A (Supporting Information), PYK2 ablation in BC cells (two independent clones of two sgRNAs; KO2, Figure 2B, and KO12, Figure S2A, Supporting Information) also reduced tumor growth by ≈40%, and the excised tumors displayed similar H&E staining of less condensed tissue (Figure S2B, Supporting Information). Importantly, the inhibitory effects on tumor growth were associated with a significantly reduced number of infiltrating macrophages as quantified by IHC analysis for F4/80 (Figure 2C; Figure S2C, Supporting Information). To confirm the IHC results on infiltrated macrophages, we performed flow cytometry analysis of size‐matched tumors (Figure S2D, Supporting Information). We adapted a protocol described by Bolli et al.,^[^ ^31^ ^]^ and defined macrophages by their CD45^+^CD11b^+^Ly6C^lo‐int^Ly6G^−^SiglecF^−^F4/80^hi^ phenotype (Figure S2F, Supporting Information). As shown in Figure 2D, a profound reduction of total macrophages was obtained in PYK2 KO tumors as well as significantly less Ly6C^hi^ monocytes (Figure 2E), which may represent a subset of myeloid‐derived suppressor cells.^[^ ^32^ ^]^

In a second model, where WT BC cells (EO771) were implanted into PYK2 KO mice (Figure 2F), we also observed a significant reduction in tumor volume (≈50% at the endpoint, *p* = 0.0004) compared to WT BC cells grown in WT background (Figure 2G), thus highlighting the impact of PYK2 ablation only within the TME. The inhibitory effects on tumor growth were accompanied by a massive decrease in the number of infiltrated macrophages as shown by F4/80 immunostaining (Figure 2H). Flow cytometry analysis of size‐matched tumors (Figure S2E, Supporting Information) supported the IHC results and showed a substantial reduction in CD45^+^CD11b^+^Ly6C^lo‐int^Ly6G^−^SiglecF^−^F4/80^hi^ macrophages (Figure 2I), while Ly6C^hi^ monocyte numbers were unchanged (Figure 2J).

Collectively, these results show that ablation of PYK2 either in BC cells or in the TME substantially reduced TAM numbers. A summary of the different mouse models and the effect of the respective PYK2 manipulations on both tumor size and macrophage infiltrates (IHC analysis) is shown in Figure 2K. These different models highlight the discrete impact of PYK2 ablation either in BC cells or the TME as well as the therapeutic potential of an effective and selective inhibitor for PYK2.

### 2.3 Ablation of PYK2 in Breast Cancer Cells Impairs CCL2 Secretion, Notch1 Signaling, and Macrophage Recruitment

**Figure 3:**
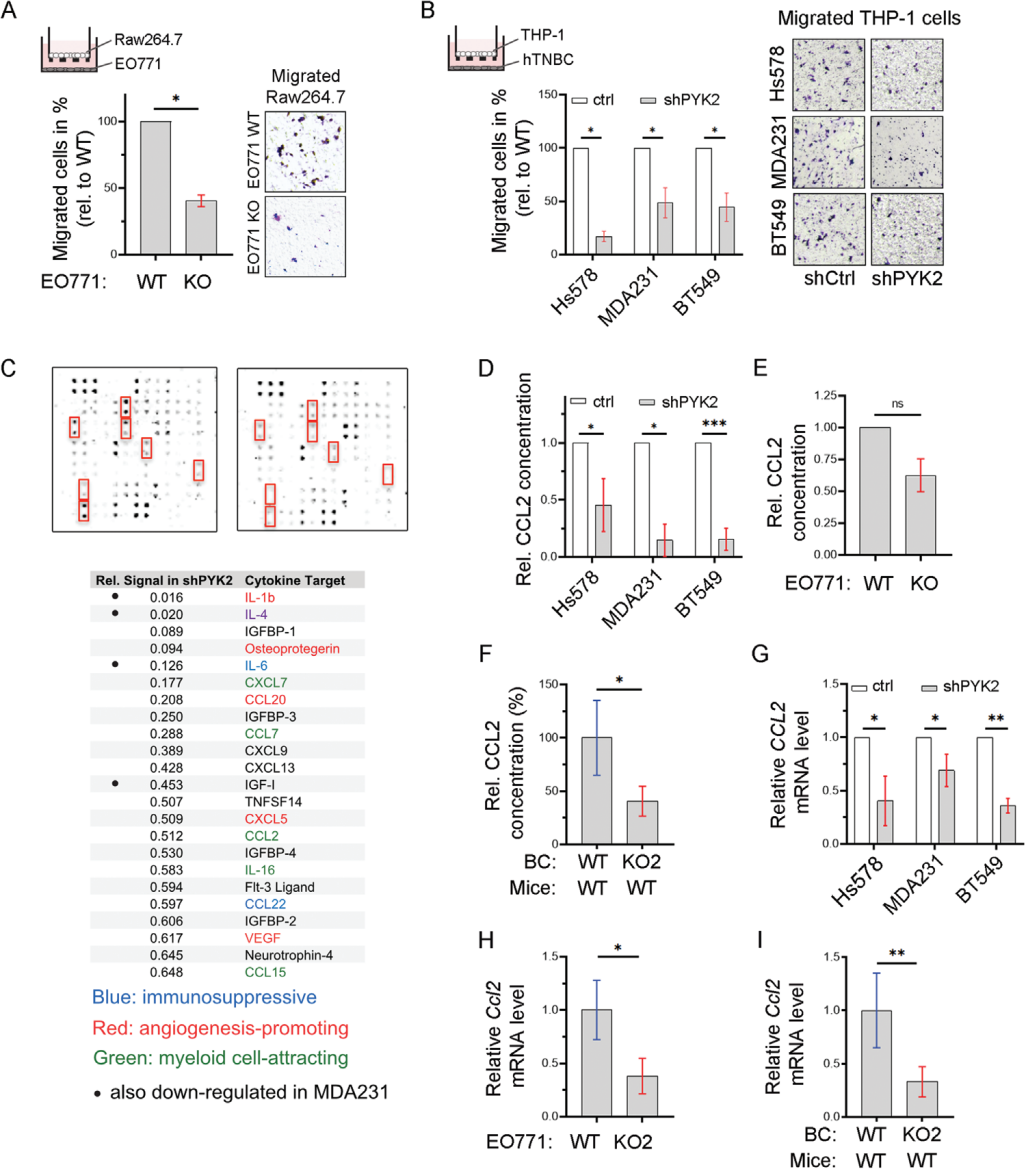
PYK2 deletion in breast cancer cells modulates secretion of CCL2 and other cytokines. A) Scheme of the transwell system with murine WT Raw264.7 macrophages in the upper chamber and control or PYK2 KO EO771 breast cancer cells in the bottom. Migration of Raw264.7 cells was quantified 4 h after seeding. Shown are mean values (ratios of KO to WT) ± SD of two independent experiments. Statistical significance was determined by one sample *t*‐test. Representative images are shown on the right. B) Schematic diagram of a transwell system with human THP‐1 monocytes in the upper chamber and control or PYK2 KD (by shRNA) human TNBC cell lines (Hs578T, MDA‐MB‐231, and BT549) in the bottom. Migration of THP‐1 was quantified 16 h after seeding. Shown are mean values (ratios of KD to control) ± SD of 2 (MDA‐MB‐231) or 3 (Hs578T, BT549) independent experiments. Statistical analysis was determined by one sample *t*‐test. Representative images are shown on the right. C) Conditioned media of control and PYK2 KD BT549 and MDA‐MB‐231 (Figure S3A, Supporting Information) cells were collected and analyzed by a Human Cytokine array (Experimental Section). Results of control and PYK2‐depleted BT549 cells are shown. Relative signal intensities of significantly downregulated secreted cytokines/chemokines from PYK2‐depleted BT549 cells relative to control cells are shown in the table. D) Levels of secreted CCL2 in conditioned media of control and PYK2 KD human TNBC cell lines (Hs578T, MDA‐MB‐231, and BT549) were assessed by ELISA. Shown are relative values in PYK2 KD as ratio of control, as obtained in two independent experiments with two samples each. Statistical significance was determined by one sample *t*‐test. E) Relative levels of CCL2 in conditioned medium of PYK2 KO (KO2) EO771 cells compared to WT control was determined by ELISA. Shown are results (ratios of KO to WT) from two independent experiments. Statistical significance was determined by one sample *t*‐test. F) Relative levels of CCL2 protein in tumor lysates from PYK2 KO (KO2) EO771 tumors (*n* = 4) compared to WT tumors (*n* = 4). Breast cancer cells were injected into WT mice and CCL2 level was assessed by ELISA. Statistical significance was determined by Welch *t*‐test. G) Relative levels of human CCL2 transcripts in control and PYK2 KD human TNBC cell lines (Hs578T, MDA‐MB‐231, and BT549) were determined by qPCR. Shown are mean values (ratios of KD to control) ± SD of 3 (Hs578T, BT549) or 5 (MDA‐MB‐231) independently generated sample groups (ratios of KD to control each). Statistical significance was determined by one sample *t*‐test. H) Relative levels of mouse Ccl2 transcripts in WT and PYK2 KO (KO2) EO771 cells were determined by qPCR. Shown are mean values ± SD of 3 (KO2) or 4 (WT) independently generated samples. Statistical significance was determined by Welch *t*‐test. I) Relative levels of mouse Ccl2 transcripts in tumors driven from WT (*n* = 5) or PYK2 KO2 (*n* = 6) EO771 cells were determined by qPCR. Statistical significance was determined by Welch *t*‐test.

The finding that PYK2 depletion in BC cells massively reduced the number of infiltrated macrophages (Figure 2C,D) implies that PYK2 is required for monocyte/macrophage attraction. Since many secreted factors, including chemokines, cytokines, and growth factors can mediate the crosstalk between tumor cells and macrophages,^[^ ^30^ ^]^ we first examined the influence of PYK2 depletion in BC cells on chemotaxis of macrophages in vitro using transwell assays. We examined both mouse and human breast cancer cell lines; the mouse system included both WT and PYK2 KO BC EO771 cells and the murine Raw264.7 macrophage line, while the human system included several TNBC cell lines (BT549, MDA‐MB‐231, Hs578T; WT and PYK2 knockdown (KD)) and the human monocytic cell line THP‐1.^[^ ^33^ ^]^ As shown in **Figure** **3**A,B, depletion of PYK2 in BC cells significantly attenuated chemotaxis of macrophages toward tumor cells, suggesting that PYK2 depletion modulates the secretome of cancer cells and consequently macrophage attraction.

To identify the relevant secreted factors, we analyzed conditioned media of WT and PYK2‐depleted human BT549 and MDA‐MB‐231 cells by cytokine array (80 Human Cytokines: AAH‐CYT‐G5; RayBiotech) (Figure 3C; Figure S3A, Supporting Information). Several cytokines known to be involved in monocyte/macrophage recruitment were substantially reduced in the supernatants of the two PYK2‐depleted TNBC cell lines, such as IL‐1*β* and IL‐6, while other chemokines (CCL2, CXCL7, CCL7, CCL15) were strongly reduced at least in BT549.^[^ ^34^, ^35^, ^36^, ^37^, ^38^ ^]^ As CCL2 plays a major role in monocyte recruitment to most solid tumors,^[^ ^34^ ^]^ we validated the results for this chemokine by ELISA using conditioned media of WT and PYK2‐depleted human TNBC cell lines (Figure 3D) as well as murine EO771 cells (Figure 3E; Figure S3B, Supporting Information). We also analyzed tumor lysates derived from WT and PYK2 KO BC cells (EO771) in WT mice (Figure 3F; Figure S3C, Supporting Information). As shown in Figure 3D–F, PYK2 depletion significantly reduced the levels of secreted CCL2 from human and mouse BC cell lines in vitro, as well as the level of CCL2 in breast tumor lysates. We then examined whether PYK2 depletion affects CCL2 transcription in human TNBC cell lines, murine EO771 BC cells, and EO771‐derived tumor tissues (Figure 3G–I; Figure S3D,E, Supporting Information) by qPCR. Significant reduction was observed in all cases analyzed, indicating that PYK2 affects CCL2 transcription.

**Figure 4:**
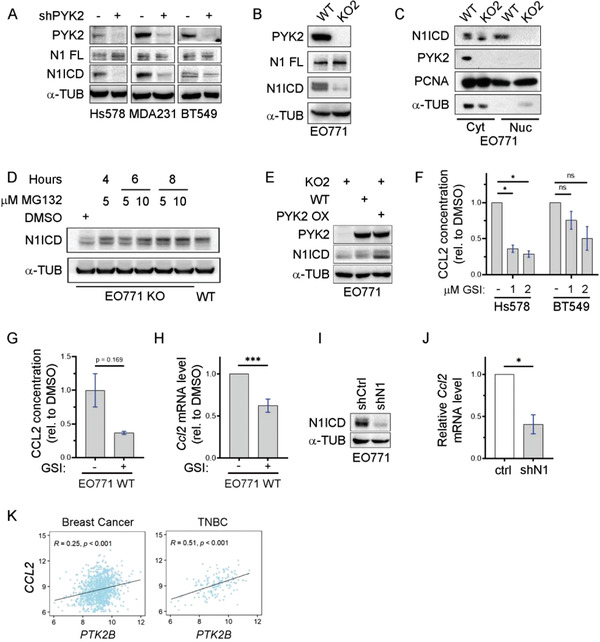
Link between PYK2, Notch1, and CCL2 in breast cancer. A,B) Protein levels of full length Notch1 (N1 FL) and N1ICD in either control or PYK2 KD (shPYK2) TNBC cell lines (A), or in control (WT) and PYK2 KO (KO2) EO771 cells (B) were assessed by Western blot. C) Western blot analysis of N1ICD in cytosolic (Cyt) and nuclear (Nuc) fractions from WT and PYK2 KO (KO2) EO771cells. D) PYK2 KO EO771 cells (KO2) were treated with the indicated concentrations of the proteasome inhibitor MG132 for the indicated time points. The protein levels of N1ICD in untreated (DMSO), MG132 treated, and control WT EO771 lysates were determined by Western blotting E) PYK2 overexpression in EO771 PYK2 KO cells restored N1ICD levels as shown by Western blot analysis using the indicated antibodies. F) Effect of gamma secretase inhibitor (GSI, LY411575) on the level of secreted CCL2 from Hs578T and BT549 TNBC cells was assessed by ELISA using conditioned media from control (−) and GSI‐treated cells (1 × 10^−6^ m, 2 × 10^−6^ m) for 24 h. Shown are relative concentration of treated samples compared to untreated control as obtained from two independent experiments. Shown are mean values (ratios of treated to untreated) ± SD. Statistical significance was determined by one sample *t*‐test. G) Relative CCL2 level in conditioned medium from EO771 WT cells treated with GSI (1 × 10^−6^ m LY411575) for 24 h, as compared to vehicle control (−). Shown are mean values (ratios of treated to untreated) ± SD from two independent experiments. Statistical significance was determined by Welch *t*‐test. H) Relative *Ccl2* mRNA levels in EO771 cells treated with GSI (1 × 10^−6^ m LY411575) for 24 h as determined by qPCR analysis. Shown are mean values ± SD of five independently generated sample groups (ratios of treated (+) to untreated (−)). Statistical significance was determined by one sample *t*‐test. I) Western blot analysis confirmed Notch1 deletion upon lentivirus‐mediated knockdown (shN1) in EO771 WT cells. J) Relative *Ccl2* mRNA levels in EO771 Notch1 knockdown cells as compared to control cells were determined by qPCR analysis. Shown are mean values ± SD of four independently generated sample groups (ratios of KD to control each). Statistical significance was determined by one sample *t*‐test. K) Correlation between *PTK2B* and *CCL2* expression in all breast cancer samples (*n* = 958) or only in TNBC samples (*n* = 138) using the TCGA datasets. Pearson's correlation coefficient (*R*) and *p*‐values are indicated.

The influence of PYK2 on CCL2 transcription (Figure 3G–I), led us to examine whether PYK2 regulates CCL2 transcription via Notch signaling, as previous studies showed that Notch1 regulates IL‐1*β* and CCL2 expression in mammary carcinoma and consequently modulates macrophage recruitment.^[^ ^39^ ^]^ We, therefore, examined the level of Notch1 protein in WT and PYK2‐depleted human TNBC cell lines (**Figure** **4**A) or murine EO771 cells (Figure 4B; Figure S4A, Supporting Information). As Notch1 activation requires proteolytic release of Notch1 intracellular domain (N1ICD) and its subsequent nuclear translocation to regulate transcription of target genes,^[^ ^40^, ^41^ ^]^ we assessed both full‐length Notch1 and N1ICD by WB. As shown in Figure 4A,B and Figure S4A (Supporting Information), PYK2 depletion had no obvious effect on full‐length Notch1, but substantially reduced N1ICD levels. Cell fractionation showed that the N1ICD was present both in the nucleus and the cytosol of WT EO771 cells, but only weakly detected in the cytosol of PYK2 KO cells (Figure 4C; Figure S4B, Supporting Information). These results suggest that PYK2 regulates either the nuclear translocation of N1ICD and/or N1ICD stability. Indeed, treatment of PYK2 KO EO771 cells with the proteasome inhibitor MG132 restored the level of N1ICD (Figure 4D; Figure S4C, Supporting Information), suggesting that PYK2 stabilizes N1ICD. Importantly, overexpression of PYK2 in PYK2 KO EO771 cells rescued N1ICD levels (Figure 4E), thus highlighting the specificity of PYK2 and excluding off‐target effects.

To further corroborate the link between Notch1 and CCL2 in the BC cell lines, we inhibited N1ICD release by the gamma secretase inhibitor (GSI) LY411575. We confirmed the inhibition by Western blotting with an antibody against N1ICD (Figure S4D, E, Supporting Information), and showed by ELISA that CCL2 secretion was reduced in both the human and murine BC cells (Figure 4F,G). GSI treatment also reduced transcription of CCL2 (Figure 4H), transcription of Notch1 itself and of the Notch target gene *Hes1* (Figure S4F, Supporting Information) in EO771 cells.

To directly demonstrate the influence of Notch1 on the *Ccl2* transcript, we depleted Notch1 by shRNA in EO771 cells (Figure 4I) and examined *Ccl2* mRNA levels by qPCR. As shown in Figure 4J, Notch1 knockdown markedly reduced *Ccl2* level (by 60%) as well as *Plau*, another Notch1 regulated factor,^[^ ^42^ ^]^ whereas *Hes1*, a common target gene of all Notch family members, was not changed, indicating that Hes1 is Notch dependent but Notch1‐independent in these cells (Figure S4G, Supporting Information).^[^ ^43^ ^]^ Importantly, we found that PYK2 gene expression (*PTK2B*) is correlated with *CCL2* (Figure 4K; Figure S4H, Supporting Information) as well as *IL1B* (Figure S4I, Supporting Information) expression in breast cancer patients, further strengthening the link between PYK2 and these Notch1‐regulated cytokines and highlighting the clinical relevance of our findings.

### 2.4 Ablation of PYK2 Only in Macrophages Is Sufficient to Reduce Tumor Growth and Macrophage Infiltration

**Figure 5:**
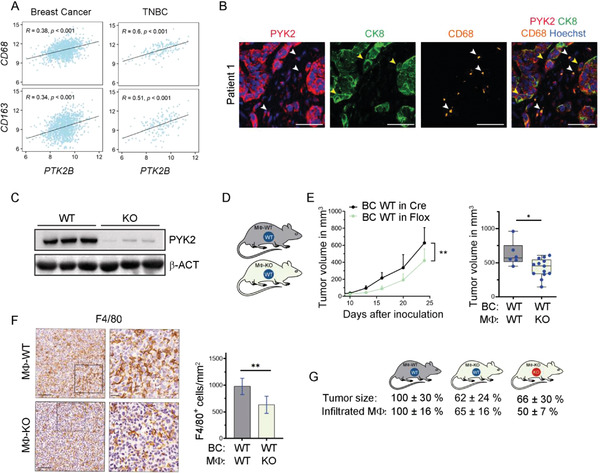
PYK2 correlates with macrophage markers in human TNBC and its macrophage‐specific ablation reduces tumor growth and TAM numbers. A) Pearson's correlation between *PTK2B* and *CD68* or *CD163* expression in all breast cancer samples (*n* = 958) or only in TNBC samples (*n* = 138) using the TCGA datasets. Pearson's correlation coefficient (*R*) and *p*‐values are indicated. B) Representative images of human TNBC sections immuno‐stained for PYK2 (red), cytokeratin 8 (CK8, green), and the macrophage marker CD68 (orange). Macrophages (white arrowheads), tumor cells (yellow arrowheads). Scale bar: 50 µm. C) Western blot analysis for PYK2 of BMDMs from Cre control (MΦ‐WT) and macrophage specific PYK2 KO (MΦ‐KO) mice. D) Schematic representation of the analyzed tumor model; EO771 WT BC cells were injected into Cre control (Mϕ‐WT) or macrophage‐specific PYK2 KO (MΦ‐KO) mice. E) Tumor growth curves over 24 days show the mean tumor volume at the indicated time points following implantation of WT EO771 cells into MΦ‐WT (*n* = 6) and MΦ‐KO (*n* = 13) mice; statistical significance was determined by two‐way ANOVA. Volume of individual tumors at day 24 is shown in the box plot; statistical significance was determined by Welch *t*‐test. F) Quantification of F4/80‐stained cells on sections from WT tumors in MΦ‐WT (*n* = 6) and MΦ‐KO (*n* = 14) mice. Representative IHC images are shown. Scale bar: 100 µm, 25 µm (for inset). G) Graphical summary of tumor volume and relative numbers of TAMs (IHC) in the different models studied. Effects were calculated as fold of control (%).

The remarkable effects of PYK2 ablation either in BC cells or in the TME on tumor growth and macrophage infiltration (Figure 2) highlight a link between PYK2 and TAMs. To demonstrate its clinical relevance, we assessed the correlation between PYK2 expression (PTK2B gene) and macrophage markers (CD68, CD163^[^ ^44^ ^]^) in human breast cancer datasets. As shown in **Figure** **5**A, *PTK2B* expression significantly correlates with the general macrophage marker *CD68* (*R* = 0.38, *p* < 0.001) as well as with the pro‐tumorigenic macrophage marker *CD163* (*R* = 0.34, *p* < 0.001) in BC patients, and even more profoundly in two independent datasets of TNBC patients (TCGA, *n* = 138; *R* = 0.6 (*CD68*), *R* = 0.51 (*CD163*); Figure 5A, GSE76124, *n* = 198; *R* = 0.38 (*CD68*), *R* = 0.26 (*CD163*); Figure S5A, Supporting Information). Moreover, examination of PYK2 protein expression in human breast cancer sections by IF analysis confirmed the expression of PYK2 protein in both breast cancer cells (Cytokeratin 8‐positive) and TAMs (CD68 positive) (Figure 5B; Figure S5B, Supporting Information).

To further characterize the functional link between PYK2 and TAMs, we examined the effects of PYK2 ablation only in macrophages on tumor growth and macrophage infiltration. To this end, we generated macrophage specific PYK2 KO mice (Cx3cr1‐Cre/PYK2^f/f^, referred to as Mϕ‐KO) by crossing PYK2^f/f^ mice with mice expressing the Cre recombinase under control of the Cx3cr1 promotor.^[^ ^45^ ^]^ First, we confirmed the KO of PYK2 in macrophages by Western blot analysis of bone marrow‐derived macrophages (BMDMs) from Mϕ‐KO compared to Cx3cr1‐Cre^tg/wt^/PYK2^wt/wt^ (Mϕ‐WT) mice (Figure 5C). Then, WT EO771 cells were orthotopically implanted into the mammary fat pad of Mϕ‐WT and Mϕ‐KO mice (Figure 5D) and tumor growth was measured over time. Tumor growth was significantly reduced in Mϕ‐KO mice compared to Mϕ‐WT mice with an average reduction of 38% after 24 days (Figure 5E). No obvious microscopic changes were observed by H&E staining of tumor sections (Figure S5C, Supporting Information). IHC analysis revealed a significant reduction of F4/80^+^ macrophages in tumors from Mϕ‐KO compared to Mϕ‐WT mice (Figure 5F).

Importantly, a decrease in tumor volume (≈34%, Figure S5E, Supporting Information) and number of infiltrated macrophages (Figure S5F, Supporting Information) was also obtained when PYK2 KO EO771 cells were orthotopically implanted into Mϕ‐KO mice (Figure S5D, Supporting Information). Figure 5G summarizes tumor sizes and macrophage numbers (IHC) for the macrophage‐specific PYK2 depletion system. The data highlight the crucial role of TAMs for breast cancer growth.

### 2.5 Defects of PYK2 Knockout Macrophages

**Figure 6:**
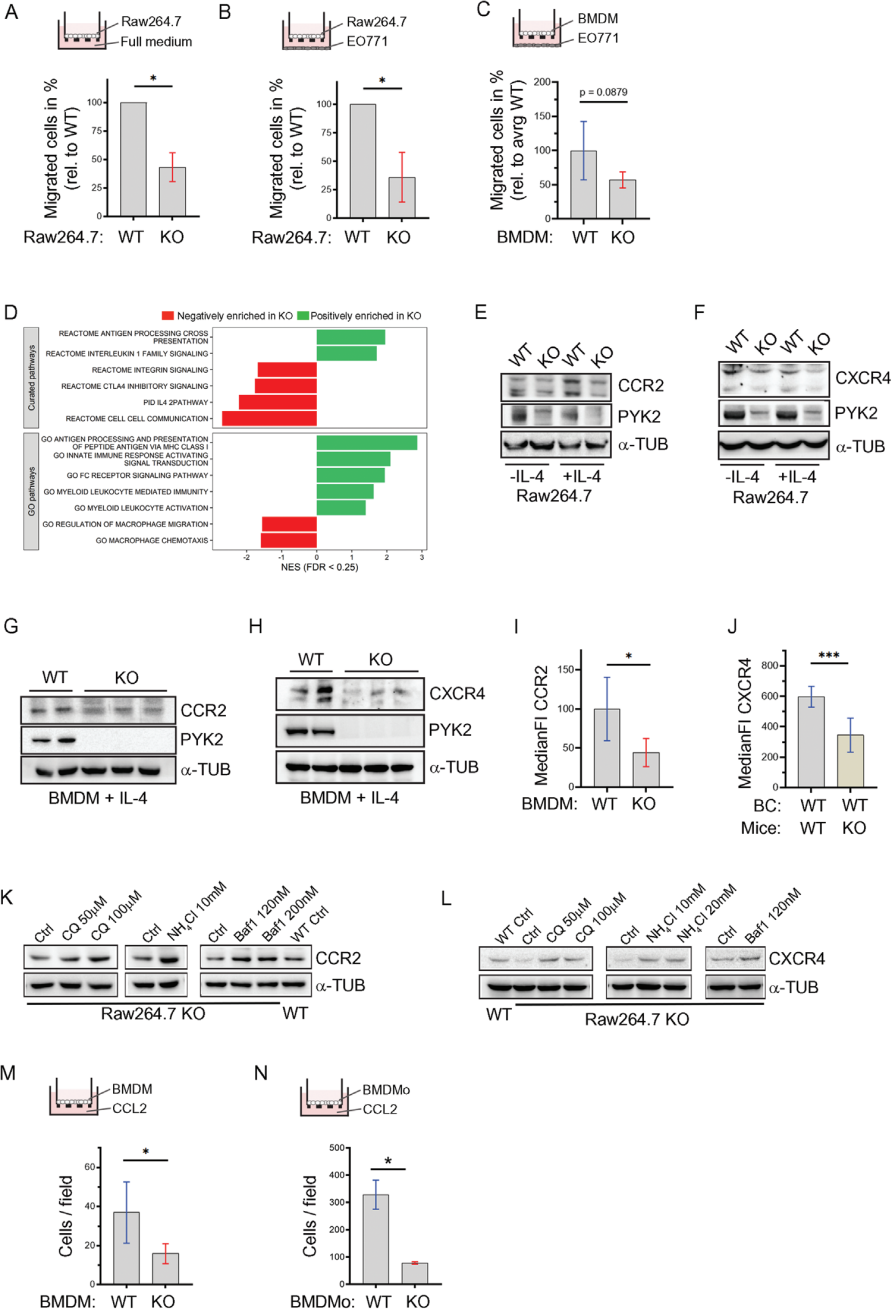
PYK2 ablation modulates the transcriptome, migration, chemotaxis, and protein levels of CCR2 and CXCR4 in macrophages. A) Migration of WT and PYK2 KO Raw264.7 cells toward full medium was assessed by transwell migration assay. Ratios of KO to WT (depicted in %) from two independent experiments are shown. Statistical significance was determined by one sample *t*‐test. B) Migration of WT and PYK2 KO Raw264.7 cells toward EO771 WT cells was assessed by transwell migration assay. Ratios of KO to WT (depicted in %) from three independent experiments are shown. Statistical significance was determined by one sample *t*‐test. C) Results of transwell migration assay of BMDMs from MΦ‐WT (*n* = 5) and MΦ‐KO (*n* = 5) mice toward EO771 cells are shown. Values are depicted as migrated cells in percentage relative to average WT value. Statistical significance was determined by Welch *t*‐test. D) Gene set enrichment analysis (GSEA) of RNA‐Seq data from WT and PYK2 KO BMDMs. Bone marrow cells were derived from WT and PYK2 KO mice (*n* = 3 each) (Experimental Section). RNA was extracted and subjected to RNA‐Seq analysis. GSEA was performed using curated signatures from the MSigDB database, as well as the gene ontology (GO) biological processes pathways. Shown is the normalized enrichment score (NES) of relevant significant (FDR < 0.25) pathways. E,F) Western blot analysis shows the levels of CCR2 (E), CXCR4 (F), and PYK2 proteins in untreated (−IL‐4) or IL‐4‐treated (+IL‐4) Raw264.7 cells (WT and PYK2 KO). G,H) Western blot analysis shows the levels of CCR2 (G), CXCR4 (H), and PYK2 proteins in IL‐4‐treated (+‐IL4) BMDMs. I) Levels of CCR2 on the surface of BMDMs from WT (*n* = 5) and PYK2 KO (*n* = 5) mice was determined by flow cytometry (Experimental Section). Median fluorescent intensity (MFI) for CCR2 was measured and MFI of the respective isotype control subtracted. Statistical significance was determined by Welch *t*‐test. J) Levels of CXCR4 on the surface of F4/80^hi^ macrophages from EO771 WT tumors in WT (*n* = 8) and PYK2 KO (*n* = 8) mice was determined by flow cytometry (Experimental Section). MFI for CXCR4 was measured and MFI of the respective isotype control subtracted. Statistical significance was determined by Welch *t*‐test. K) Western Blot analysis of CCR2 levels in Raw264.7 cells (WT and KO) treated with the indicated lysosomal inhibitors (CQ = chloroquine, Baf1 = bafilomycin) for 12 h. L) Western Blot analysis of CXCR4 levels in Raw264.7 cells (WT and PYK2 KO) treated with the indicated lysosomal degradation inhibitors as described in (J). M) Results of transwell migration assay of BMDMs from WT (*n* = 5) and PYK2 KO (*n* = 5) mice toward medium containing 0.5% heat inactivated FBS and 20 ng mL^−1^ murine CCL2. Statistical significance was determined by Welch *t*‐test. N) Results of transwell migration assay of BMDMo (bone marrow derived monocytes enriched fraction) from WT (*n* = 3) and PYK2 KO (*n* = 3) mice toward medium containing 0.5% heat inactivated FBS and 20 ng mL^−1^ murine CCL2. Statistical significance was determined by Welch *t*‐test.

As shown in Figure 5, selective ablation of PYK2 in macrophages significantly attenuated tumor growth and concomitantly reduced TAM numbers, implying that ablation of PYK2 in macrophages results in cell autonomous defects and/or impairs their crosstalk with tumor cells. To address these possibilities, we first examined the influence of PYK2 ablation in macrophages on cell migration by modified Boyden chamber migration assays. As shown in **Figure** **6**A, migration of PYK2 KO Raw264.7 cells toward complete medium (10% fetal bovine serum (FBS)) was markedly reduced compared to WT Raw264.7 cells, suggesting a cell‐autonomous effect of PYK2. In addition, we observed defects in migration of PYK2 KO Raw264.7 cells (Figure 6B) as well as PYK2 KO BMDMs toward WT BC cells (EO771) (Figure 6C), suggesting effects of PYK2 on BC–macrophage communication.

To better characterize the phenotype of PYK2 depleted macrophages, we performed bulk RNA sequencing of BMDMs derived from WT and PYK2 KO mice. Gene expression of PYK2 KO BMDMs compared to WT control is shown in Table S1 of the Supporting Information, which confirmed the decrease in *PTK2B* expression (Figure S6A, Supporting Information). Gene set enrichment analysis (GSEA) highlighted changes in key pathways including cell–cell communication, cell migration, chemotaxis, and several pathways related to macrophage function, including processing of external signals, macrophage immunity and antigen presentation, as well as the IL‐4 pathway (Figure 6D). These pathways of macrophage‐autonomous defects are consistent with previous reports demonstrating impaired macrophage migration, cell polarization,^[^ ^20^ ^]^ and phagocytic activity in PYK2 KO mice.^[^ ^46^ ^]^

The RNA‐seq data (Figure 6D) together with the chemotaxis results (Figure 6A–C) and our previous studies demonstrating the broad influence of PYK2 on different cell surface receptors^[^ ^16^, ^19^, ^47^ ^]^ suggest that PYK2 may affect key chemotactic receptors as well as IL‐4Rα. Hence, we first examined the effect of PYK2 KO on two major chemoattractant receptors in macrophages, CCR2 and CXCR4,^[^ ^48^ ^]^ by WB. As shown, KO of PYK2 markedly reduced the protein levels of these two receptors in Raw264.7 cells (Figure 6E,F) and in BMDMs (Figure 6G, H). Importantly, we also found reduced surface expression of CCR2 on BMDMs (Figure 6I) and of CXCR4 on TAMs (Figure 6J) using flow cytometry analysis. We further showed that PYK2 depletion enhanced lysosomal degradation of these receptors, as lysosomal inhibitors (chloroquine, NH_4_Cl, bafilomycin (Baf1)) rather than the proteasomal inhibitor MG132 could restore the receptor levels (Figure 6K, L).

The reduced surface expression of CCR2 on BMDMs was accompanied with significantly reduced chemotaxis of PYK2 KO BMDMs toward media containing recombinant CCL2 in the lower chamber (Figure 6M). Moreover, *ex vivo* analysis of monocytes enriched from BM (BMDMo) further confirmed that PYK2 depletion impaired migration toward CCL2 (Figure 6N; Figure S6B, Supporting Information). Altogether, these findings demonstrate that PYK2 ablation reduced the levels of key chemotactic receptors and consequently of monocyte trafficking. These observations highlight the dual role of PYK2 in regulating CCL2‐CCR2 signaling through concurrent influence on CCL2 release from breast cancer cells and of CCR2 protein levels in macrophages.

### 2.6 PYK2 Modulates IL‐4Rα Signaling in Macrophages and Influences Their Pro‐tumorigenic Phenotype

**Figure 7:**
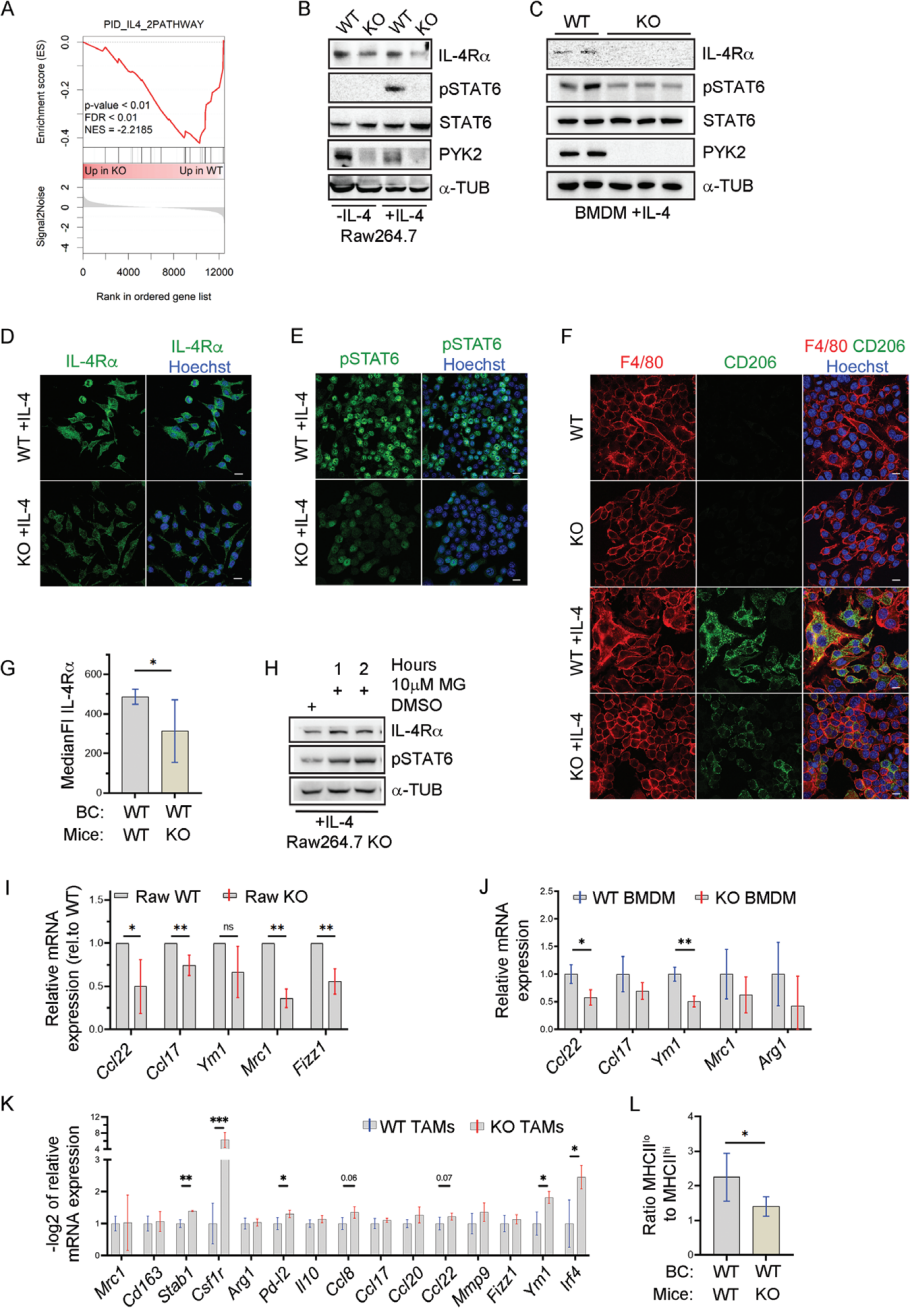
PYK2 depletion affects IL‐4Rα levels, STAT6 phosphorylation, macrophage polarization, and pro‐tumorigenic markers. A) GSEA of the IL‐4 pathway reveals its relative enrichment in WT compared to PYK2 KO BMDMs. B,C) Western blot analysis shows the levels of IL‐4Rα, STAT6, pSTAT6, and PYK2 proteins in untreated (−IL‐4) or IL‐4‐treated (+‐IL4) Raw264.7 cells (WT and KO) (B), and IL‐4‐treated BMDMs (C). D) Representative immunofluorescence (IF) images of IL‐4‐treated WT and PYK2 KO Raw264.7 cells immunostained for IL‐4Rα. Scale bar: 10 µm. E) Representative immunofluorescence (IF) images of IL‐4‐treated WT and PYK2 KO Raw264.7 cells immunostained for pSTAT6. Scale bar: 10 µm. F) Representative immunofluorescence (IF) images of non‐ and IL‐4‐treated WT and PYK2 KO Raw264.7 cells double immunostained for F4/80 and CD206. Scale bar: 10 µm. G) Levels of IL‐4Rα on the surface of F4/80^hi^ macrophages sorted from tumors of EO771 WT (*n* = 8) and PYK2 KO (*n* = 8) mice was determined by flow cytometry (Experimental Section). MFI for IL‐4Rα was measured and MFI of the respective isotype control subtracted. Statistical significance was determined by Welch *t*‐test. H) IL‐4‐treated PYK2 KO Raw264.7 cells were treated with the proteasome inhibitor MG132 (MG, 10 × 10^−6^ m) for the indicated time points. The protein levels of IL‐4Rα and pSTAT6 in untreated (DMSO) and MG132 treated Raw264.7 cell lysates were determined by Western blotting. I) Expression of pSTAT6 target genes in IL‐4‐treated PYK2 KO Raw267.4 cells relative to WT control was assessed by qPCR. Shown are mean values of 4–5 independently generated sample groups (ratios of KO to WT each). Statistical significance was determined by one sample *t*‐test for each target. J) Expression of pSTAT6 target genes in IL‐4‐treated WT (*n* = 3) and PYK2 KO (*n* = 3) BMDMs was assessed by qPCR. Statistical significance was determined by Welch *t*‐test for each target. K) Expression of polarization marker genes in TAMs sorted from EO771 WT tumors induced in WT and PYK2 KO mice was assessed by qPCR. Shown are −log2 values ± SD of relative mRNA expression (ΔCt) in PYK2 KO TAMs to WT control (*n* = 3–6). Statistical significance was determined by Welch *t*‐test for each target gene. L) Ratio of MHCII^low^ to MHCII^high^ macrophages in WT tumors of WT and KO mice. Percentages of MHCII^high^ and MHCII^low^ macrophages were determined by flow cytometry and the ratio calculated subsequently. Statistical significance was determined by Welch *t*‐test.

As mentioned, the RNA‐seq analysis indicated that the IL‐4 pathway was significantly altered in PYK2 KO BMDMs (Figures 6D and **7**A). This pathway is critical for induction of a pro‐tumorigenic phenotype.^[^ ^49^ ^]^ To validate the impact of PYK2 on this pathway, we assessed the level of IL‐4 receptor (IL‐4Rα) and of pSTAT6, a downstream effector of the IL‐4R pathway,^[^ ^50^ ^]^ in control and PYK2 KO Raw264.7 and BMDMs. The pro‐tumorigenic phenotype was induced by IL‐4 treatment (Experimental Section). As shown in Figure 7B, KO of PYK2 in Raw264.7 cells markedly reduced the level of IL‐4Rα in steady‐state conditions or in response to IL‐4 treatment, and robustly reduced the phosphorylation of STAT6 in IL‐4‐treated macrophages. Likewise, the levels of IL‐4Rα and pSTAT6 were significantly reduced in IL‐4‐induced BMDMs from Mϕ‐KO compared to Mϕ‐WT mice (Figure 7C). These results were further corroborated by IF analysis for IL‐4Rα (Figure 7D) in WT and PYK2 KO Raw264.7 cells, as well as by IF staining for pSTAT6 (Figure 7E) and its downstream target CD206 (Figure 7F). Importantly, significantly lower levels of surface IL‐4Rα (MFI) were also detected by flow cytometry analysis of TAMs from WT tumors in PYK2 KO compared to the WT background (Figure 7G). Blocking the proteasomal degradation in the Raw264.7 KO cells restored IL‐4Rα and pSTAT6 levels (Figure 7H), suggesting that PYK2 protects IL‐4Rα from degradation similar to its protective effects on other receptors, including Notch1 (Figure 4). Collectively, these results suggest that PYK2 regulates IL‐4Rα levels and consequently the IL‐4R‐pSTAT6 pathway.

Numerous studies have shown that activation of IL‐4Rα induces macrophage polarization into an M2‐like phenotype in vitro, and that M2‐like macrophages are implicated in pro‐tumorigenic effects in vivo.^[^ ^51^ ^]^ We, therefore, examined the influence of PYK2 ablation on the expression of M2‐like markers. WT and PYK2 KO Raw264.7 cells (Figure 7I) or BMDMs (Figure 7J) were treated with IL‐4 and the mRNA expression levels of pSTAT6 target genes including *Mrc1, Arg1*, *Fizz1, Ym1*, *Ccl22*, and *Ccl17* were assessed.^[^ ^52^ ^]^ As shown, ablation of PYK2 reduced the expression levels of all these genes, often reaching significance. To evaluate the effects of PYK2 on the macrophage phenotype in the TME, we used fluorescence‐activated cell sorting to isolate TAMs from size‐matched breast tumors in WT and PYK2 KO background (Figure S7A,B, Supporting Information), and analyzed mRNA expression of several pro‐tumorigenic polarization markers (Figure 7K). As shown, PYK2 KO TAMs expressed reduced levels of the following pro‐tumorigenic markers: *Stab1, Pd‐l2 (Pdcd1lg2), Ym1, Irf4* (all *p* < 0.05) and *Il10, Ccl8, Ccl17, Ccl20, Ccl22, Mmp9*, *and Fizz1* (all *p* > 0.05). Furthermore, we found a robust decrease of *Csf1r* mRNA, which plays a crucial role in monocyte and macrophage survival.^[^ ^53^ ^]^ In addition, we stratified mature F4/80^high^ macrophages by MHC class II surface expression and found a significant reduction in MHCII^low^ pro‐tumorigenic macrophages in PYK2 KO tumors, while the MHCII^high^ (anti‐tumorigenic) population was unchanged (Figure S7C,D, Supporting Information). Consequently, the MHCII^low^ to MHCII^high^ ratio was significantly reduced upon PYK2 KO (Figure 7L), thus, further suggesting that PYK2 ablation decreases the pro‐tumorigenic phenotype.

Together, these results suggest that PYK2 ablation not only inhibited macrophage polarization in vitro, but most importantly also reduced their pro‐tumorigenic phenotype in the TME.

### 2.7 PYK2 Ablation Reduces Tumor Angiogenesis

**Figure 8:**
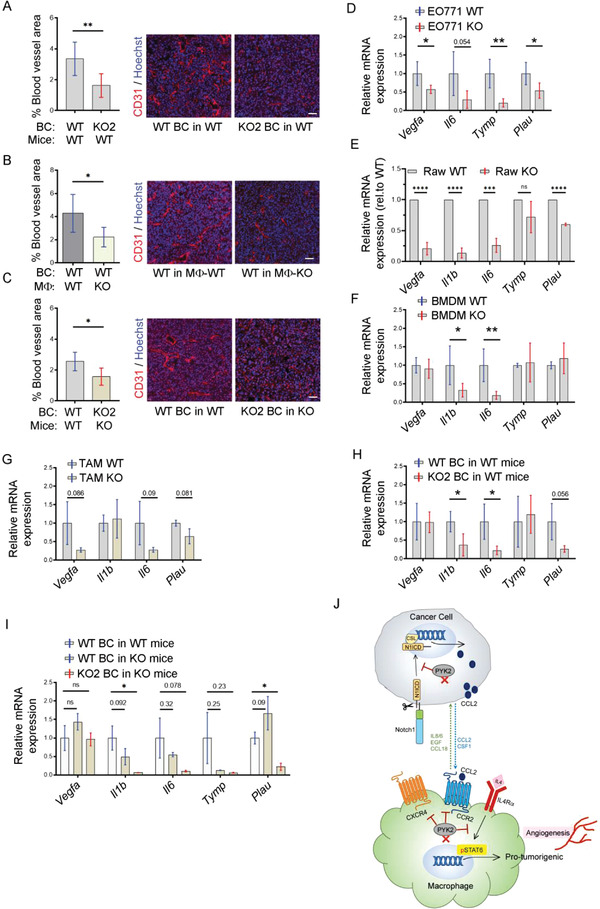
PYK2 depletion reduces tumor angiogenesis. A–C) Quantification and representative IF images of blood vessel area in tumors induced by injection of (A) WT EO771 (*n* = 6) or PYK2 KO2 EO771 (*n* = 7) cells into WT mice (B) WT EO771 cells into either MΦ‐WT (*n* = 6) or MΦ‐KO (*n* = 8) mice; and (C) WT EO771 cells into WT mice (*n* = 5) or PYK2 KO2 EO771 cells into PYK2 KO mice (*n* = 6). Statistical significance was determined by Welch *t*‐test. Scale bar: 50 µm. D–I) Expression of angiogenesis‐related genes in WT (*n* = 5) and PYK2 KO (*n* = 5) EO771 cells (D), in WT (*n* = 5) and PYK2 KO (*n* = 5) Raw264.7 cells (E), in WT (*n* = 5) and PYK2 KO (*n* = 5–6) BMDMs (F), and TAMs sorted from EO771 WT tumors induced in WT (*n* = 4) and PYK2 KO (*n* = 3) mice (G), in EO771 WT (*n* = 4) and KO (*n* = 5–6) tumors induced in WT mice (H), and in EO771 WT tumors induced in WT (*n* = 3–4) and KO (*n* = 4) mice and in EO771 KO tumors induced in KO (*n* = 3) mice (I). Statistical significance was determined by Welch *t*‐test for (D), (F), (G), and (H); by one sample *t*‐test for (E); and by Brown‐Forsythe and Welch ANOVA with Dunnett's T3 multiple comparison test for (I). J) Model showing the multifaceted roles of PYK2 in breast cancer–macrophages communication, macrophages recruitment, and polarization. In breast cancer cells, PYK2 stabilizes N1ICD, which regulates CCL2 transcription and consequently CCL2 release. CCL2 secreted from cancer cells is required for recruitment of monocytes/macrophages through their CCR2 receptor. In addition to the CCL2/CCR2 axis, macrophage recruitment is regulated by other chemotactic receptors, such as CXCR4, and by cell autonomous migratory properties, which are also affected by PYK2 deficiency. Ablation of PYK2 in macrophages also reduced the level of IL‐4Rα, the main receptor needed for pro‐tumorigenic polarization, and robustly reduced STAT6 activation. Consequently, transcriptions of STAT6 target genes are decreased, leading to less pro‐tumorigenic TAMs, reduced angiogenesis, and tumor growth. CSL (CBF‐1, Suppressor of Hairless, Lag‐2), a transcriptional regulator and N1CD binding protein.

The profound effects of PYK2 ablation on TAM numbers (Figures 1, 2, and 5F; Figure S5F, Supporting Information) together with the known effects of TAMs on tumor angiogenesis^[^ ^54^ ^]^ led us to assess the influence of PYK2 ablation either in the BC cells and/or in the TME on angiogenesis using the described different mouse models. CD31 was used as a blood vessel marker to stain tumor sections and evaluate total blood vessel area (Experimental Section). The results were compared between the different tumor models of PYK2 ablation and corresponding WT control. As shown, the blood vessel area was significantly reduced when PYK2 was knocked out either in the BC cells (**Figure** **8**A), only in the macrophages (Figure 8B), or both in the BC and the entire TME (Figure 8C).

To gain insight into the underlying mechanism of reduced blood vessel area in the PYK2 ablated mouse models, we examined the mRNA levels of key factors known to regulate different stages of angiogenesis/vessel formation, including *Vegfa, Il1b*, *Tymp1, Plau*, *and Il6*,^[^ ^55^, ^56^ ^]^ by qPCR. Reduced expression levels of these proangiogenic factors were obtained in PYK2‐ablated EO771 cells (Figure 8D), Raw264.7 (Figure 8E), BMDMs (Figure 8F), and TAMs (Figure 8G). Analysis of tumor samples indicated that ablation of PYK2 either in the BC cells (Figure 8H), in the TME (Figure 8I), or both in the BC and in the TME (Figure 8I) also reduced the expression of the examined proangiogenic factors. These results suggest that PYK2 can modulate tumor angiogenesis through several mechanisms, and its ablation in the BC cells and/or in the TME can reduce the levels of certain proangiogenic factors and attenuate angiogenesis.

Collectively, our studies uncovered novel mechanisms mediated by PYK2 to control breast cancer–macrophage communication and introduced a unique approach to demonstrate the impact of TAMs on breast cancer progression (Figure 8J).

## 3 Conclusion

The pro‐tumorigenic influence of TAMs on breast cancer progression suggests that targeting of TAMs could be a beneficial therapeutic strategy.^[^ ^15^ ^]^ Indeed, suppression of monocyte recruitment to the TME or inhibition of TAM polarization into pro‐tumorigenic macrophages successfully inhibited breast cancer growth in different preclinical models.^[^ ^4^, ^14^, ^57^ ^]^ In this study we show that depletion of the nonreceptor tyrosine kinase PYK2 markedly reduced the number of macrophages in breast tumors (Figures 1, 2, and 5F; Figure S5F, Supporting Information), and concurrently reduced tumor angiogenesis (Figure 8), and tumor growth (Figures 1E and 2B,G; Figure S2A, Supporting Information; Figure 5E; Figure S5E, Supporting Information).

By using different genetic mouse models and ablation of PYK2 in the BC cells, the entire TME, or selectively in macrophages, we could systematically dissect the discrete influence of PYK2 on BC cells and macrophages alone, and on the BC–macrophage crosstalk signaling. These analyses together with transcriptomic profiling of BMDMs from WT and PYK2 KO mice (Figure 6D), secretome profiling of PYK2‐depleted TNBC cells (Figure 3C; Figure S3A, Supporting Information), and mechanistic studies (Figures 3, 4, 6, and 7), suggest that PYK2 ablation impairs macrophage recruitment and polarization through cell‐autonomous mechanisms and by tumor–macrophage CCL2/CCR2/CXCR4 and IL‐4/IL‐4R crosstalk signaling.

Inhibition of tumor growth was observed when PYK2 was ablated either in the BC cells (Figure 2B; Figure S2A, Supporting Information), in the TME (Figure 2G), or selectively in macrophages of the TME (Figure 5E), demonstrating the striking influence of TAMs on tumor growth, and the critical role of PYK2 in macrophage–breast cancer communication. KO of PYK2 only in macrophages was sufficient to inhibit tumor growth by ≈35% (Figure 5E), while KO of PYK2 in BC cells inhibited tumor growth by ≈40%. Nevertheless, KO of PYK2 in both BC and in the TME (Figure 1E) or both in BC and in macrophages (Figure S5E, Supporting Information) had no additive influence on tumor growth (Figure 2K). This unexpected phenotype might be related to the regulatory role of PYK2 in BC–macrophage/monocyte communication, which are usually mediated by receptor–ligand interaction, such as CCL2–CCR2. Hence, inhibition of either ligand secretion or receptor expression could be sufficient to impair receptor downstream signaling, similarly to the effect of ligand and receptor coinhibition.

We previously showed that PYK2 regulates the levels of different cell surface receptors including HER3, cMET, EGFR, AXL, and certain ligands such as IL‐8 in breast cancer cells.^[^ ^16^, ^19^, ^47^, ^58^ ^]^ Other studies demonstrated its effects on secretion of proinflammatory cytokines from macrophages, such as IL‐1*β* and IL‐18^[^ ^59^ ^]^ or IL‐6, TNF*‐α*, and IL‐12.^[^ ^60^ ^]^ Interestingly, PYK2 was found to regulate inflammatory response in the gut via direct binding and phosphorylation of IRF5 (Interferon regulating factor 5).^[^ ^60^ ^]^ Here we show that PYK2 ablation inhibits CCL2 secretion by BC cells (Figure 3D,E) and CCR2 expression in macrophages (Figure 6E, G, I), thereby impairing CCL2–CCR2 signaling and consequently TAM recruitment. Indeed, previous reports demonstrated the inhibitory effects of CCL2 neutralizing antibodies on breast cancer growth, metastasis, and TAM recruitment.^[^ ^61^ ^]^ We found that PYK2 regulates *Ccl2* transcription in BC cells (Figure 3F,G,H) through the Notch1 pathway by stabilizing the protein level of N1ICD (Figure 4A–C; Figure S4A,B, Supporting Information), consistent with previous reports demonstrating the influence of Notch on *Ccl2* transcription in breast cancer cells and TAM recruitment in mouse models.^[^ ^39^ ^]^ Although the underlying mechanism remains to be determined, it could be that PYK2 regulates N1ICD degradation by sequestering the ubiquitin ligase NEDD4‐1, which was shown to interact with PYK2^[^ ^16^ ^]^ and also to ubiquitinate Notch in *Drosophila* and skeletal muscle.^[^ ^62^ ^]^

Importantly, CCL2 can also bind to CCR2–CXCR4 heterodimers.^[^ ^63^ ^]^ CXCR4 was also reduced in PYK2 KO macrophages (Figure 6F,H, J), and is known to play a role in cell migration, suggesting that PYK2 regulates TAM recruitment through both cell autonomous‐dependent mechanisms and tumor–macrophage communication. Indeed, GSEA analysis of BMDMs RNA‐seq data showed significant downregulation of macrophage migration, chemotaxis and integrin signaling (Figure 6D). The RNA‐seq data also highlighted the effect of PYK2 on the IL‐4 signaling pathway (Figure 7A), and further analysis of PYK2 KO macrophages showed a substantial decrease in IL‐4Rα levels and STAT6 phosphorylation in response to IL‐4 stimulation (Figure 7B–E,G). STAT6 is a common downstream effector of IL‐4R and IL‐13R signaling, and its phosphorylation is crucial for M2 polarization.^[^ ^50^ ^]^ Phospho‐STAT6 regulates the transcription of M2‐characteristic genes including *Mrc1, Arg1, Ym1, Mrc1, Ccl17*, and *Ccl24*,^[^ ^64^ ^]^ and its inhibition attenuates breast cancer growth and enhances immunosuppressive responses of macrophages.^[^ ^50^, ^65^ ^]^ Indeed, we found significant effects of PYK2 ablation on macrophage polarization in vitro (Figure 7I,J), on the expression of pro‐tumorigenic factors (Figure 7K) as well as on the MHCII^low^ subpopulation of TAMs (Figure S7C, Supporting Information), thus demonstrating anti‐tumorigenic effects of PYK2 ablation. These results are in agreement with recent reports on the influence of exosomal PYK2 and its binding Rab22a‐NeoF1 fusion protein on M2 polarization and lung metastasis of osteosarcoma.^[^ ^66^ ^]^

Our finding that PYK2 regulates levels of key cell surface receptors including IL‐4R, CXCR2, CXCR4, and Notch1 (Figures 4, 6, and 7) and consequently, modulates critical signaling pathways in BC–TAM communication, highlights its therapeutic potential and the clinical implications of our studies. Indeed, CXCR4 blockade significantly reduced chemotaxis of pro‐tumorigenic macrophages in oral squamous cell carcinoma^[^ ^67^ ^]^ and several CXCR4 antagonists, as well as of CCR2 and CSF1R, are currently in clinical trials.^[^ ^68^ ^]^ Nevertheless, targeting of PYK2 alone might not be sufficient for cancer therapy, whereas combining PYK2 targeting with other therapeutic agents could be beneficial for subsets of breast cancer patients. Previous studies suggest that cotargeting of PYK2 and EGFR could be beneficial for basal‐like patients with high EGFR levels,^[^ ^16^ ^]^ and other studies suggest that immunosuppressive activity of TAMs is associated with upregulation of PD‐L1 and immune escape in TNBC.^[^ ^15^ ^]^ Our finding that PYK2 inhibition reduced the number of TAMs and their pro‐tumorigenic phenotype suggests that PYK2 inhibition may sensitize BC to anti‐PDL1 blockade. Indeed, previous studies showed that co‐inhibition of PYK2 and FAK enhanced the anti‐tumor efficacy of an anti‐PD‐1 immune checkpoint blockade in colorectal tumors,^[^ ^23^ ^]^ and that PYK2, but not FAK, is required for macrophage recruitment into the TME of PTEN‐null glioblastoma multiforme (GBM) to support tumor growth. In this GBM model, PTEN deficiency regulates lysyl oxidase expression, which functions as a macrophage chemoattractant via activation of the integrin *β*1‐PYK2 axis.^[^ ^69^ ^]^ Hence, development of a highly potent, selective inhibitor for PYK2 could have a therapeutic impact, especially since PYK2 and its most related kinase FAK have overlapping, but still distinct functions,^[^ ^70^, ^71^ ^]^ particularly in immune cells including macrophages.

TAMs are known for their positive influence on tumor angiogenesis.^[^ ^49^ ^]^ We found that PYK2 depletion in the cancer cells or macrophages reduced the mRNA expression of several proangiogenic factors (*Vegfa, Il1b, Il6, Tymp*, and *Plau*) (Figure 8). Importantly, reduced CCL2 and IL‐1*β* secretion was also reported to influence angiogenesis^[^ ^55^, ^72^ ^]^ suggesting that PYK2 ablation can affect angiogenesis through different routes.

Overall, we show that PYK2 regulates TAMs in a breast cancer model, and provide evidence that ablation of PYK2 only in macrophages is sufficient to attenuate tumor growth (Figure 5E), tumor angiogenesis (Figure 8B), and to reduce TAM numbers (Figure 5F). These remarkable observations not only highlight the important role of PYK2, but also show, in a unique manner, the critical impact of TAMs on breast cancer progression. Hence, our studies unveil pleiotropic effects of PYK2 on key signaling cascades that modulate macrophage recruitment, polarization, and tumorigenic properties, and highlight its clinical implications in cancer therapy.

## 4 Experimental Section

### Cell Culture

Human embryonic kidney HEK293T cell line, human triple negative breast cancer cell lines (MDA‐MB‐231, BT549, Hs578T) as well as the human acute monocytic leukemia THP‐1 cell line were originally obtained from ATCC. The mouse mammary tumor cell line EO771 and the murine macrophage Raw264.7 WT and PYK2 KO^[^ ^46^ ^]^ cell lines were kindly provided by R. Alon (Weizmann Institute of Science, IS) and by Christof R. Hauck (University of Konstanz, Konstanz, Germany), respectively. The murine fibroblast cell line L929 was obtained from S. Jung (Weizmann Institute of Science, Rehovot, Israel). All cell lines, except L929, were grown in RPMI. L929 cells were grown in DMEM. EO771 medium was supplemented with HEPES (20 × 10^−3^ m, pH 7.4). Cells were grown in medium containing 10% FBS and penicillin–streptomycin (100 U mL^−1^; 0.1 mg mL^−1^). Heat‐inactivated FBS was used for THP‐1, Raw264.7 cells, and BMDMs. Cell lines were routinely checked for the presence of mycoplasma by PCR.

### Conditioned Media Preparation

Cells were grown to 80% of confluency, washed with PBS, and then incubated for 24 h in serum‐free medium. Conditioned medium was collected, filtered through a 0.22 µm filter and either used directly for secretome analysis or ELISA or snap‐frozen in liquid nitrogen and stored at −80 °C. L929‐conditioned medium was prepare as previously described.^[^ ^73^ ^]^ In brief, L929 cells were grown in a 10 cm dish until confluency. Subsequently, the cells were split with trypsin and grown in 25 mL DMEM (10% heat‐inactivated FBS, penicillin–streptomycin (100 U mL^−1^; 0.1 mg mL^−1^) in 175 cm^2^ flasks for 2 days until confluency. 20 mL of fresh medium were added after 2 days, and the conditioned medium was collected 10 days later. The medium was filtered (0.22 µm), snap‐frozen in liquid nitrogen and stored at −20 °C until use.

### Bone Marrow Derived Macrophage Isolation

Bone marrow cells were obtained by flushing the femurs and tibias of mice with DMEM/F‐12. After erythrocyte lysis with Red blood cell lysis buffer (1.55 m NH_4_Cl, 0.92 m NaHCO_3_, 1.0 × 10^−3^ m EDTA) for 5 min at RT, cells were washed twice with ice‐cold PBS and bone marrow cell suspension was cultured at 4 × 10^6^ cells mL^−1^ in BMMF medium (DMEM/F‐12 medium with 10% heat‐inactivated FBS (v/v), 100 U mL^−1^ penicillin, 100 µg mL^−1^ streptomycin, 2 × 10^−3^ m l‐glutamine, 5 × 10^−3^ m HEPES pH 7.4, 2.5 µg mL^−1^ fungizone, 0.5 mg mL^−1^ gentamycin sulfate, and 20% L929‐conditioned DMEM (v/v) as a source of M‐CSF) for 7 days. After 3 days in culture, fresh BMMF medium was added. For polarization, BMDMs were subsequently incubated for additional 2 days with IL‐4 and IL‐13 (20 ng mL^−1^ each; Table S2, Supporting Information) in BMMF medium.

### Bone Marrow Derived Monocyte Enrichment

Bone marrow cell suspensions were obtained from mice according to the method described above. To enrich monocytes, a two‐step process of Ficoll density gradient centrifugation and subsequent magnetic‐activated cell sorting (MACS) using positive selection of CD115^+^ cells was applied. The BM cell suspension in 4 mL PBS was carefully pipetted onto 4 mL Ficoll‐Paque (room temperature) in a 15 mL tube. After centrifugation at 2200 rpm (RT, no braking) for 15 min the upper layer was removed before the interphase (buffy coat) was collected. The latter was washed with 12 mL PBS and centrifuged for 7 min at 4 °C and 1400 rpm. The pellet was resuspended in 50 µL MACS‐buffer (2% FBS, 1 × 10^−3^ m EDTA) containing 1:100 CD115‐Biotin antibody (Miltenyi Biotec) and incubated for 15 min on ice in the dark. After washing with 5 mL MACS buffer, centrifugation at 1400 rpm at 4 °C and removal of the supernatant, 30 µL streptavidin beads (Miltenyi Biotec) in 270 µL MACS buffer were added to the cells. The samples were incubated for 20 min on ice in the dark with shaking every 5 min, washed again, resuspended in 1 mL MACS buffer, filtered through 70 µm mesh, and loaded onto the magnet‐fixed MACS column, which was prewashed with 3 mL MACS buffer. After the solution ran through, the run‐through solution was mixed with the 3 mL MACS buffer from the column wash and loaded again. The column was then washed with 6 mL MACS buffer. Finally, the MACS column was removed from the magnet and placed on top of a 15 mL tube. 5 mL MACS buffer was added and pushed with the stamp through the column. This step was repeated once before the cells were centrifuged at 1400 rpm, 4 °C for 7 min and then resuspended in 0.5 mL MACS buffer to be counted.

### Mice

PYK2*^−^* ^/−^ (PYK2 KO) mice^[^ ^26^ ^]^ as well as PYK2^f/f^ mice^[^ ^25^ ^]^ were established and provided by JA. Girault. Homozygous PYK2^f/f^ mice were mated with homozygous Cx3cr1^cre^ mice,^[^ ^45^ ^]^ generated and provided by S. Jung, to obtain offspring heterozygous for *PTK2B* and hemizygous for *Cx3cr1‐Cre*. These Cx3cr1‐Cre^tg/wt^/PYK2^f/wt^ mice were then crossed to generate Cx3cr1‐Cre^tg/wt^/PYK2^f/f^, which were finally subjected to experiments (females only). To maintain that line, Cx3cr1Cre^tg/wt^_PYK2^f/f^ (Mϕ‐KO) were crossed continuously. Mice hemizygous for *Cx3cr1‐Cre* (Cx3cr1‐Cre^tg/wt^) (Mϕ‐WT) served as controls and were obtained by breeding homozygous Cx3cr1^cre^ mice with wild type C57BL/6 OlaHsd (ENVIGO, Israel). All offspring were genotyped from tail or ear biopsy using the primers listed in Table S3 of the Supporting Information. All mice were in C57BL/6 background. All mice were maintained in a pathogen‐free facility, and all animal procedures were approved by the Weizmann Institute's Animal Care and Use Committee (IACUC, investigator accreditation number WIS‐060).

### Tumor Experiments

Wild‐type or Mϕ‐WT C57BL/6 mice as well as PYK2 KO mice or Mϕ‐KO were orthotopically injected with 0.5 × 10^6^ EO771 (WT or PYK2 KO) cells in 50 µL PBS. The cells were implanted in the 3rd mammary fat pad of 8–10‐week‐old female mice. Tumor size was measured every 3–4 days intervals with a caliper and the volume was calculated using the equation: *V*  = [length  ×  (width)^2^]/2. When tumors reached the size of ≈0.6–1 cm^3^, animals were euthanized, and tumors were removed. Per experiment, at least three mice per group were injected with tumor cells, experiments were repeated at least twice, and at least six mice were used per group.

For the flow cytometric analyses of tumor composition using size‐matched tumors, models associated with PYK2 ablation (EO771 PYK2 KO in WT background or WT EO771 cells in PYK2 KO background) were injected 3 or 4 days, respectively, prior to their corresponding WT control. Tumors were analyzed on day 18 from first injection of the KO models.

### DNA Constructs, Lentivirus Production and Infection and Knockout by CRISPR/Cas9

Lentiviral vectors (pLKO.1‐puro) encoding shRNAs of human PYK2 and mouse Notch1 were used as previously described.^[^ ^19^ ^]^ Lentivirus purification and infection was performed as described previously.^[^ ^74^, ^75^ ^]^ Knockdown was confirmed by Western blotting. Representative results obtained with human PYK2 shRNA TRCN0000231519 and mouse Notch1 shRNA TRCN0000025935 are shown in the manuscript. To knockout PYK2 by CRISPR/Cas9, EO771 were transfected with pSpCas9(BB)‐2A‐GFP (pX458) plasmid (Addgene #48138) encoding the Cas9 enzyme, an EGFP expression cassette, and a guide RNA targeting either exon 1 (5′‐GGGCCCCCCAGAGCCCATGG‐3′) or exon 2 (5′‐GCTGCACCCACAGATGACCG‐3′) of the *PTK2B* gene.^[^ ^46^ ^]^ Transfection was performed by jetPRIME (Polyplus Transfections) according to the manufacturer's protocol. 24 h after transfection, cells harboring the Cas9‐GFP encoding plasmid were isolated by flow cytometry‐associated cell sorting (BD FACSAriaII) and single clones were plated in 96‐well plates. After 2–3 weeks, propagated clones were analyzed for PYK2 expression by Western blotting (Figure S1A, Supporting Information), and DNA sequencing was performed in selected lines to verify deletion (data not shown). Lentivirus (pHAGE‐PGK‐IRES‐Hygro‐W) encoding HA‐tagged wild‐type human PYK2 was used to overexpress PYK2.^[^ ^19^ ^]^

### Analysis of BC Datasets

Correlation between *PTK2B* expression and other genes in breast cancer patients was carried out using the Cancer Genomic Atlas (TCGA, *n* = 956 patients, among which 138 are TNBC patients) and an additional dataset of 198 TNBC patients (taken from GSE76124^[^ ^76^ ^]^). Pearson's correlation was performed in R.

### RNA Extraction and Real‐Time PCR Analysis

Total RNA was extracted using TRI Reagent (Sigma‐Aldrich). cDNA was generated from 1 µg of RNA using the High‐Capacity cDNA Reverse Transcription Kit (Applied Biosystems, Foster City, CA, USA). Subsequently, cDNA was subjected to quantitative real‐time PCR using SYBR Green I as a fluorescent dye (Roche) according to the manufacturer's instruction. Real‐time PCR analysis was performed on an ABI StepOnePlus 7500 Real‐Time PCR system (Applied Biosystems, Invitrogen). All experiments were normalized to *Rps29* (mouse) and *ACTB* (human) RNA levels. Real‐time PCR primers were designed using Primer designing tool from NCBI, NIH and were calibrated before use. The primer sequences are listed in Table S4 of the Supporting Information.

### RNA‐Seq and GSEA

RNA was extracted from BMDMs seven days after isolation using TRI Reagent as described above. RNA quality was assessed using Agilent 4200 TapeStation System (Agilent Technologies, Santa Clara, CA). RNA‐seq libraries were generated by applying a bulk adaptation of the MARS‐seq protocol, as described previously.^[^ ^77^ ^]^ Libraries were sequenced by the Illumina Novaseq 6000 using SP mode 100 cycles kit (Illumina). Mapping of sequences to the genome and generation of the count matrix was performed by the UTAP pipeline (Weizmann Institute). Libraries normalization, filtration of low count genes and discovery of differentially expressing genes was performed using the edgeR package in R. GSEA was performed using GSEA software (Broad). RNA‐seq was performed in three biological replicates. RNA‐seq data is available in GEO accession No. GSE193168.

### Immunoblot Analysis

Cells or tumor tissue samples were homogenized in lysis buffer (0.5% Triton‐X‐100, 50 × 10^−3^ m HEPES pH 7.5, 100 × 10^−3^ m NaCl, 1 × 10^−3^ m MgCl_2_, 5 × 10^−3^ m EGTA, 10% glycerol, 50 × 10^−3^ m NaF, 0.5 × 10^−3^ m NaVO_3_, 20 × 10^−3^ m *β*‐glycerol phosphate, 1 × 10^−3^ m phenylmethylsulfonyl fluoride, 10 µg mL^−1^ leupeptin, and 10 µg mL^−1^ aprotinin). Samples were vortexed, incubated on ice for 15 min, and then centrifuged at 14 000 rpm for 15 min at 4 °C. Protein concentration of the samples supernatants was determined by Bradford assay (Bio‐Rad, Hercules, CA). Equal amounts of proteins were resolved by 8% SDS–polyacrylamide gel electrophoresis, and then transferred to nitrocellulose membranes and blotted with corresponding primary antibodies (Table S5, Supporting Information) as described previously.^[^ ^74^ ^]^ The proteins of interest were visualized in a ChemiDoc MP imaging system (Bio‐Rad) using ECL (Bio‐Rad).

### Subcellular Fractionation

Nuclear and cytoplasmic extracts were prepared from ≈6 × 10^6^ cells. Cells were washed with PBS and then incubated with 100 µL CE buffer (10 × 10^−3^ m HEPES, 60 × 10^−3^ m KCl, 1 × 10^−3^ m EDTA, 1 × 10^−3^ m DTT, 1 × 10^−3^ m PMSF, adjusted to pH 7.6, 0.075% (v/v) NP‐40) for 3 min on ice. The samples were centrifuged at 1000 rpm for 4 min at 4 °C and supernatant containing the cytoplasmic fraction was transferred to a new tube. The pellet containing the nuclei was washed twice with 100 µL CE‐buffer without NP‐40 and pelleted by 4 min microcentrifugation at 1000 rpm at 4 °C. 50 µL of NE buffer (20 × 10^−3^ m Tris HCl, 420 × 10^−3^ m NaCl, 1.5 × 10^−3^ m MgCl2, 0.2 × 10^−3^ m EDTA, 1 × 10^−3^ m PMSF, 25% (v/v) glycerol, adjusted to pH 8) and 3.5 µL of 5 m NaCl were added. Samples were incubated for 10 min on ice and vortexed every 2 min. Both nuclear and cytoplasmic fraction were centrifuged at 14 000 rpm for 10 min, before the supernatants were transferred to new tubes. Glycerol was added to the cytoplasmic fraction to 20% before analyzing the extracts by 8% SDS–polyacrylamide gel electrophoresis.

### Flow Cytometry of Cells from Tumor Tissue

Eight size‐matched tumors per group were chopped and then digested by shaking for 45 min in 5 mL digestion buffer (1 mg mL^−1^ of collagenase A and 150 µg mL^−1^ of Hyaluronidase, 1% penicillin–streptomycin in RPMI) at 37 °C to obtain single cell suspensions. Tumor fragments were dissociated with a 10 mL pipet several times during the 45 min incubation. Viable cells were counted after filtering the digested samples through 70 µm Falcon cell strainers. Loosely attached cells were collected by washing the strainer with 2 mL PBS. Tumor cells were collected by centrifuging the cell suspension for 5 min at 300 g at 4 °C. Cells were washed and suspended in PBS. 5 × 10^6^ cells in PBS were subsequently stained in 100 µL FACS buffer (sterile PBS with 3% BSA) against all extracellular target proteins for 30 min on ice, before the cells were washed in FACS buffer, and then fixed. Cells were fixed using the BD Cytofix/Cytoperm Kit according to the manufacturer's instructions. Briefly, the cells were fixed in 50 µL Cytofix for 20 min on ice. The cells were then washed and resuspended in 300–500 µL FACS buffer for analysis, which was carried out on a BD Biosciences FACSAriaIII. Further analyses were performed using FlowJo V10 (TreeStar). For this experiment a live/dead staining was not included.

### TAM Isolation Using FACS

Five or six tumors per group were isolated and digested as described above, and 20 × 10^6^ cells in PBS were subsequently stained with LIVE/DEAD Fixable Blue for 30 min at RT in the dark. After washing the cells with FACS buffer (sterile PBS with 3% BSA), TrueStain FcX in FACS buffer was used to block the cells for 20 min at RT. Cells were subsequently incubated with fluorophore‐conjugated antibodies targeting CD45, CD11b, F4/80, Ly6C, Ly6G, and SiglecF on ice in the dark for 30 min and then washed with FACS buffer, centrifuged at 300 g for 5 min, resuspended in 500 µL FACS buffer and sorted using the BD Biosciences FACSAriaIII. CD45^+^CD11b^+^Ly6C^lo‐int^Ly6G^−^SiglecF^−^F4/80^hi^ cells were sorted into RPMI + 10% FBS. Then, RNA was isolated using TriReagent as described above.

### Immunofluorescence Staining of Cells

Immunofluorescence (IF) staining was performed as previously described.^[^ ^19^ ^]^ Briefly, cells were grown in 24‐well plates on glass coverslips and cultured for 48 h in the presence or absence of IL‐4 (20 ng mL^−1^). After washing with PBS, cells were fixed with 2% (IL‐4Rα) or 4% paraformaldehyde (PFA) (CD206, F4/80) in PBS for 20 min at room temperature or with cold Acetone for 20 min at −20 °C (pSTAT6). The cells were then incubated at RT for 10 min in 0.1 m glycine in PBS, followed by 30 min incubation in blocking buffer (10 × 10^−3^ m Tris, pH 7.5, 150 × 10^−3^ m NaCl and either 10% goat serum (IL‐4Rα, pSTAT6) or 10% horse serum (CD206), 2% bovine serum albumin and either 0.1% Triton‐X‐100 (IL‐4Rα, CD206, F4/80) or 0.3% Triton‐X‐100 (pSTAT6)). Primary antibody (dilution prepared in corresponding blocking buffer) was applied over night at 4 °C. Cells were washed three times with PBS and subsequently incubated with fluorescence‐labeled secondary antibodies for 1 h in the dark. Cells were washed three times with PBS and incubated for 5 min with 2 ng  µL ^−1^ Hoechst 33342, washed again and mounted on microscopic slides using mounting media (10 × 10^−3^ m phosphate buffer, pH 8.0, 16.6% w/v Mowiol 4–88, and 33% glycerol). A confocal laser‐scanning microscope (LSM 800; Carl Zeiss) equipped with a 63 Å/1.4 oil differential interference contrast M27 objective lens (Plan Apochromat; Carl Zeiss) was used to analyze the IF staining. Images were acquired using the Zeiss ZEN 2.5 (blue edition) software.

### Immunohistochemistry and Immunofluorescence of Tumor Sections

Human tissue samples of invasive breast cancer cases were obtained with institutional review board approval (Ethik‐Kommission Fachbereich Medizin der Goethe‐Universität Frankfurt, DE) and written informed consent from patients undergoing surgical resection at the Department of Gynecology and Obstetrics at the Goethe‐University in Frankfurt am Main (DE). TNBC samples were identified according to standard pathological criteria, including estrogen receptor, progesterone receptor, and HER2 status.

Paraffin‐embedded formalin fixed human and mouse mammary tumors sections were de‐paraffinized and rehydrated as previously described.^[^ ^78^ ^]^ Sections were then incubated with H_2_O_2_ (1% v/v) in methanol solution for 30 min to quench endogenous peroxidases (for IHC). Antigen retrieval was then performed by microwave irradiation for 10 min in citric acid (pH 6.1 for CD31, CD3, Ly6C, *α*SMA, NK1.1) or in Tris‐EDTA buffer (10 × 10^−3^ m Tris‐HCl and 1 × 10^−3^ m EDTA; pH 9.0 for PYK2, CD68, CK8). For F4/80 antibody, Trypsin digestion was used as antigen retrieval. The sections were incubated at 37 °C for 10 min covered with Trypsin solution (0.1% CaCl_2_, 50 × 10^−3^ m Tris, 150 × 10^−3^ m NaCl, pH 7.8, 0.1% Trypsin, warmed to 37 °C) prior to endogenous peroxidase quenching. Sections were then blocked 1.5 h at RT using normal horse serum (20% (v/v) in PBS, 0.1% Triton‐X‐100) prior to incubation with primary antibody overnight at 4 °C. After incubation with biotinylated secondary antibody, sections were washed and treated with ABC reagent (Vector labs). Sections were then developed with DAB reagent (Sigma‐Aldrich) and counterstained with hematoxylin for 30 s. Stained slides were scanned on a whole slide scanner (Pannoramic SCAN II, 3DHISTECH, Budapest, Hungary). To quantify macrophages, F4/80‐stained cells were counted on 6–10 random fields from each tumor slide using ImageJ 1.53g or Adobe Photoshop 2018.

For the IF staining, tissue paraffin embedded sections were stained according to the protocol above with slight modifications. No quenching of endogenous peroxidases was needed. After primary antibody incubation and washing with PBS three times for 5 min, sections were incubated with fluorescence‐labeled secondary antibodies for 1.5 h in the dark. After washing the sections three times with PBS, Höchst staining was performed for 1 min and sections mounted with Mowiol mounting media.

### Proliferation Assay (MTT)

Cells were plated in triplicates in normal medium in 96‐well plates. Cell viability was assessed at the indicated time points by incubating cells with medium containing MTT (3‐(4,5‐dimethylthiazolyl‐2)−2,5‐diphenyltetrazolium bromide) solution (0.5 mg mL^−1^; Sigma) for 2 h at 37 °C. Cells were then lysed in 100 µL lysis buffer (Isopropanol containing 0.4% NP‐40 in 0.04 mol L^−1^ HCl), and absorbance was measured using ELISA microplate reader (Corning, NY, USA) at 570 nm with a 620 nm reference wavelength.

### Transwell Cell Migration

Human or murine cancer cells were plated in the lower compartment of a 24‐transwell plate (BD Bioscience, San Jose, CA) at densities that reached 100% confluency within 24 h. The medium was then removed, the cells were washed twice with PBS and then incubated with fresh medium (without FBS for human breast cancer cells and THP‐1; with 0.5% heat‐inactivated FBS for murine cancer cells and Raw264.7). Macrophages were seeded in the upper chambers (2.5 × 10^4^ THP‐1 in serum‐free RPMI (pretreated with 200 × 10^−9^ m phorbol‐12‐myristate‐13‐acetate (PMA) for 16 h; 7.5 × 10^4^ Raw264.7 cells in RPMI with 0.5% heat‐inactivated FBS; 2 × 10^5^ BMDMs). For measuring BMDMs migration toward CCL2, 5 × 10^5^ BMDMs were plated in the insert of 24‐well plate and migrated toward medium containing 0.5% heat‐inactivated FBS and 20 ng mL^−1^ murine CCL2 for 3.5 h. Macrophage migration through the membrane (5 µm pore size) was determined 16 h (THP‐1), 4 h (Raw 264.7), 8 h (BMDMs) or 3.5 h (BMDMs to CCL2) later, by fixing the cells for 20 min in PFA (1% PFA, 20% MeOH in PBS) containing Methyl Violet stain (0.3%). Inserts were washed with tap water after removing nonmigrating cells with cotton swabs from the upper side of the filter and dried overnight. Migrated macrophages were photographed with a binocular microscope and pictures were photomerged. 6–8 pictures were taken from the photomerged image and quantified manually by counting single cells using Adobe Photoshop or automatically using ImageJ by measuring stained pixels. Averages from the 6–8 pictures of migrated cells are depicted.

### ELISA

Levels of secreted CCL2 in conditioned medium of cultured cells or tumor lysates were analyzed following the manufacturer's instructions using MCP‐1 ELISA and CCL2 ELISA kits (Peprotech, Table S2, Supporting Information). Results were normalized to cell number (for conditioned medium) or protein concentration (tumor lysate) as determined by Bradford assay (Bio‐Rad).

### Secretome Array

Secretion of cytokines/chemokines from human TNBC cell lines (BT549 and MDA‐MB‐231) were evaluated by a semiquantitative Human Cytokine G‐Series G5 Antibody Array and fluorescent detection (RayBiotech, #AAH‐Cyt‐G5, Inc., Norcross, GA), according to the manufacturer's instructions. The glass chip was dried completely and sent to RayBiotech where the chip was scanned using the laser scanner Innopsys’ InnoScan using the Cy3 channel (excitation frequency = 532 nm). After subtracting the background signal and normalizing to the positive control signals from the chip, obtained fluorescence intensity data of cell samples were compared by calculating the ratio of PYK2‐depleted cell signal to control cell signal.

### Statistics

All data are shown as mean ± SD of at least two representative experiments. Statistical significance was determined by a two‐tailed Student's *t*‐test with Welch's correction, one sample *t*‐test or Brown‐Forsythe and Welch ANOVA with Dunnett's multiple comparison test, as indicated in the figure legends. Statistical significance of tumor growth was determined by mixed‐effects model (in case of missing values for an animal) or 2‐way ANOVA, as indicated in the legends. All statistical analyses were performed using Graphpad Prism9 (^∗^ *p* < 0.05; ^∗∗^ *p* < 0.01; ^∗∗∗^ *p* < 0.001).

## Conflict of Interest

The authors declare no conflict of interest.

## Author Contributions

A.‐K.M. and U.A.K. contributed equally to this work. A.‐K.M. involved in conception and design of the study, development of methodologies, acquisition, analysis, and interpretation of data, design, and organization of figures. U.A.K. involved in conception and design of the study, development of methodologies, design and performance of in vivo experiments, data acquisition, analysis and interpretation, design and organization of figures. S.T. involved in design, performance, and analysis of flow cytometry experiments and TAM sorting. Y.V. analyzed the RNA‐seq data and the correlation between different genes expression in BC patient datasets, reviewed the raw data of the manuscript. H.M.R. was involved in IF analysis shown in Figure 6C–E. J.‐A.G. generated the PYK2*^−^* ^/−^ and PYK2^f/f^ mice, provided excellent suggestions, and reviewed the manuscript. N.B.C. provided excellent suggestions throughout the study, reviewed the flow cytometry data, and critically reviewed the manuscript. A.M. provided reagents and valuable suggestions related to Notch experiments and critically reviewed the manuscript. S.J. generated the *Cx3cr1‐Cre* mice, provided excellent suggestions throughout the study, reviewed the flow cytometry data, and critically reviewed the manuscript. S.L. designed and supervised the study, contributed to the analysis and interpretation of the data, ensured financial support, involved in figure preparation, and wrote the manuscript.

## Supporting information

Supporting Information

Supporting Information

## Data Availability

The data that support the findings of this study are available from the corresponding author upon reasonable request.
