# Supplementary material for: Mouse Modeling Dissecting Macrophage–Breast Cancer Communication Uncovered Roles of PYK2 in Macrophage Recruitment and Breast Tumorigenesis
Source: Adv Sci (Weinh). 2022 Jan 29;9(9):2105696. doi: 10.1002/advs.202105696 (PMC8948556; doi:10.1002/advs.202105696)
Supplement: Supplementary file 1 — Supporting Information [file ADVS-9-2105696-s002.pdf]

ADVANCED SCIENCE
Open Access

Supporting Information

for *Adv. Sci.*, DOI 10.1002/advs.202105696

Mouse Modeling Dissecting Macrophage–Breast Cancer Communication Uncovered Roles of PYK2 in Macrophage Recruitment and Breast Tumorigenesis

*Anna-Katharina Müller, Ulrike A. Köhler, Sébastien Trzebanski, Yaron Vinik, Harsha Mohan Raj, Jean-Antoine Girault, Nir Ben-Chetrit, Antonio Maraver, Steffen Jung and Sima Lev**

ADVANCED SCIENCE
Open Access

Supporting Information

for *Adv. Sci.*, DOI: 10.1002/advs.202105696

Mouse modeling dissecting macrophage-breast cancer communication uncovered roles of PYK2 in macrophage recruitment and breast tumorigenesis

*Anna-Katharina Müller, Ulrike A. Köhler, Sébastien Trzebanski, Yaron Vinik, Harsha Mohan Raj, Jean-Antoine Girault, Nir Ben Chetrit, Antonio Maraver, Steffen Jung and Sima Lev**

Supplementary Figures

Figure S1. Analysis of PYK2 expression and its effects on breast cancer cells

(A) Western blot showing the level of PYK2 protein in different EO771 clones transfected with pX458 plasmids encoding two different gRNAs targeting PYK2, gRNA_Exon1 (clones 1,2,4,5) or gRNA_Exon2 (clones 6,7,10,12). Clones 2 and 12 were confirmed to be PYK2 knockout lines by DNA sequencing and used for further studies.

(B) Representative images of H&E staining of WT and PYK2 KO (KO2 BC cells in PYK2 KO mice) tumor sections. Scale Bar, 50 μm.

(C) Representative images of immunohistochemical staining for PYK2 of WT and PYK2 KO (KO2 BC cells in PYK2 KO mice) tumor sections. Scale Bar, 50 μm.

(D) PYK2 deletion has no effect on proliferation of EO771 cells *in vitro*. $5x10^3$ WT, PYK2 KO2 and KO12 cells were plated in 96-well plates. MTT assay was performed for four consecutive days. Results of 2 independent experiments are shown. Statistical analysis was done by 2-way ANOVA with Dunnett's multiple comparison.

(E-I) Representative IF images of breast tumor sections driven from WT C57BL/6 mice injected with EO771 cells. The tumor sections were immuno-stained for PYK2 (red), and the T cell marker CD3 (green) (E), scale bar, 20 μm; the monocyte marker Ly6C (green) (F), scale bar, 10 μm; the fibroblast marker αSMA (green) (G), scale bar, 20 μm; the endothelial cell marker CD31 (green) (H), scale bar, 25 μm; the NK cell marker NK1.1 (green), (I), scale bar, 20 μm.

Figure S2: Tumor growth and infiltrating macrophages in the different PYK2 mouse models.

(A) Tumor volumes at day 24 of WT (WT/WT, n=6) and PYK2 KO (KO12) cells in WT mice (KO12/WT, n=10). Statistical significance was determined by Welch t test. The depicted WT samples are the same as in the graph of Figure 2B.

(B) Representative images of H&E staining of WT (WT/WT) tumor sections and PYK2 KO (KO2 and KO 12 BC cells) in WT C57B/L6 background (KO2/WT). Scale Bar, 50 μm.

(C) Numbers of F4/80-positive cells in tumors from WT/WT (n=6, the same as in Figure 2C) and KO12/WT (n=5) were determined by IHC analysis. Representative IHC pictures are shown. Scale Bar, 50 μm. Statistical significance was determined by Welch t test. The depicted WT samples are the same as in the graph of Figure 2C.

(D, E) Volume of tumors analyzed by flow cytometry: WT/WT and KO/WT (D), and WT/WT and WT/KO (E). Size-matched tumors were obtained by injecting KO tumors/mice 3 days prior to WT tumors/mice.

(F) Gating strategy for the identification of macrophages and monocytes in mammary tumors.

Figure S3: PYK2 depletion in breast cancer cells regulates cytokines release and macrophage chemotaxis.

(A) Human Cytokine array (Methods) was performed with conditioned media of control and PYK2 KD MDA-MB-231 cells. Shown in the table are relative signal intensities of significantly

down-regulated secreted factors from PYK2-depleted MDA-MB-231 cells compared to control cells.
(B) Relative levels of CCL2 in conditioned medium of PYK2 KO (KO12) EO771 cells as ratio of WT control was determined by ELISA. Shown are results from 2 independent experiments. Statistical significance was determined by one sample t test.
(C) Analysis of relative levels of CCL2 protein in tumors lysates of EO771 WT (n=4) and KO12 (n=2) cells injected into WT mice was determined by ELISA. Shown are results from 2 independent experiments. Statistical significance was determined by Welch t test.
(D) Level of mouse *Ccl2* transcript in PYK2 KO (KO12, n=4) relative to WT (n=4) EO771 cells was analyzed by qPCR. Shown are mean values ± SD of 4 independently generated samples. Statistical significance was determined by Welch t test.
(E) Level of mouse *Ccl2* transcript in PYK2 KO (KO12) tumors (n=5) relative to WT EO771 tumors (n=5) was analyzed by qPCR. Shown are mean values ± SD (5 repeats). Statistical significance was determined by Welch t test.
As experiments were performed simultaneously with EO771 KO2 and KO12, the WT samples in graphs of figures B, C, D, E are the same as in Figures 3E, F, H, I respectively.

Figure S4: Link between PYK2, Notch1 and CCL2 in breast cancer.
(A) Protein levels of full length Notch1 (N1 FL) and Notch1 ICD (N1ICD) in control (WT) and PYK2 KO (KO2, KO12) EO771 cells were assessed by Western blot. Equal protein loading was confirmed by immunoblotting with α-tubulin (α-TUB). * Figure S4A includes an overlapped portion of Figure 4B, but here we included KO12 in addition to KO2.
(B) Western blot analysis of N1ICD in cytosolic (Cyt) and nuclear (Nuc) fractions from WT and PYK2 KO (KO2 and KO12) EO771cells.
(C) PYK2 KO EO771 cells (KO12) were treated with the indicated concentrations of the proteasome inhibitor MG132 for the indicated time points. The protein levels of N1ICD in untreated (DMSO), MG132 treated, and control WT EO771 lysates were determined by Western blotting.
(D) The influence of GSI (LY411575, 1 μM, 2 μM; 24 h) on N1ICD protein levels in TNBC cell lines (Hs578, BT549) was assessed by Western blot.
(E) The influence of GSI (LY411575, 1μM, 24 h) on N1ICD protein level in EO771 cells was assessed by Western blot.
(F) Relative *Notch1 and Hes1* mRNA levels in EO771 cells treated with GSI (LY411575, 1μM, 24 h), relative to untreated control was determined by qPCR. Shown are mean values ± SD of 5 independently generated sample groups (ratios of treated to untreated each). Statistical significance was determined by one sample t test.
(G) Relative *Hes1* and *Plau* mRNA levels in EO771 cells with a lentivirus-mediated knockdown of Notch1 relative to control cells was determined by qPCR analysis. Shown are mean values ± SD of 4 independently generated sample groups (ratios of KD to control each). Statistical significance was determined by one sample t test.
(H) Correlation between *PTK2B* and *CCL2* expression in TNBC samples (n=198) using the GSE76124 dataset. Pearson's correlation coefficient (R) and p-values are indicated.
(I) Correlation between *PTK2B* and *IL1B* expression in BC samples of TCGA (n=956), TNBC samples of TCGA (n=138), and in the GSE76124 dataset (n=198). Pearson's correlation coefficient (R) and p-values are indicated.

Figure S5: PYK2 expression in human breast cancer and effects of macrophage specific PYK2 ablation.

(A) Pearson's correlation between *PTK2B* and *CD68* or *CD163* expression in TNBC patients (n=198) using the GSE76124 dataset. Pearson's correlation coefficient (R) and p-values are indicated.

(B) Representative images of human TNBC sections (different patients from Figure 5B) double immunostained for PYK2 (red) and the macrophage marker CD68 (green). Macrophages (white arrowheads), tumor cells (yellow arrowheads). Scale Bar, 100 μm.

(C) Representative images of H&E-stained tumor sections of EO771 WT tumors in Mϕ-WT and Mϕ-KO mice. Scale bar 50 μm.

(D) Schematic representation of the analyzed tumor model; WT or PYK2 KO EO771 cells were orthotopically injected into Mϕ-WT or Mϕ-KO mice.

(E) Tumor growth curves over 24 days show the mean tumor volume at the indicated time points following implantation of WT and PYK2 KO EO771 cells into Mϕ-WT (n=6) and Mϕ-KO (n=6) mice, respectively; statistical significance was determined by 2-way ANOVA. Volumes of individual tumors at day 24 is shown in the box plot; statistical significance was determined by Welch t test for individual time points. Error bars indicate mean values ± SD. The depicted WT samples are the same as in the graph of Figure 5E.

(F) Quantification of F4/80-stained cells on sections of EO771 WT tumors in Mϕ-WT (n=6) and EO771 KO tumors in Mϕ-KO (n=6) mice. Representative IHC images are shown. Scale Bars, 100 μm, 25 μm (for insert). Statistical significance was determined by Welch t test. Error bars indicate mean values ± SD. The depicted WT samples are the same as in the graph of Figure 5F.

Figure S6: PYK2 KO verification for RNAseq and flow cytometric analysis of monocytes purified by Magnetic-activated cell sorting.

(A) RNAseq was performed on RNA extracted from BMDM of WT and PYK2 KO mice (n=3 each). Differential expression of PYK2 between WT and KO macrophages in RNASeq is shown.

(B) Flow cytometric analysis of BM-derived cell suspension before and after positive enrichment of $CD115^+$ cells. Cells were stained against CD11b and Ly6C to determine enrichment levels of $CD11b^+Ly6C^{int\text{-}hi}$ monocytes.

Figure S7: Flow cytometry associated cell sorting of TAMs and stratification with MHCII

(A) Gating strategy for flow cytometry associated cell sorting of TAMs from EO771 WT tumors induced in WT and PYK2 KO mice, respectively.

(B) Tumor volumes of size-matched EO771 WT tumors induced in WT and PYK2 KO mice. Size-matched tumors were obtained by injecting KO mice 3 days prior to WT mice.
Statistical significance was determined by Welch t test.

(C-D) Flow cytometry analysis of WT/WT (n=8) and WT/KO (n=8) tumors depicted as percentages of single cells. Error bars indicate mean values ± SD. $F4/80^{hi}MhcII^{low}$ (C) and $F4/80^{high}MhcII^{high}$ (D) macrophages stratified from $F4/80^{high}$ macrophages. Statistical significance was determined by Welch t test.

Table S2. Reagents

Reagent	Company	Catalogue #
Hoechst 33342	Invitrogen	H3570
IL4	Peprotech	214-14
IL13	Peprotech	210-13
CCL2	Peprotech	900-k126
Collagenase A	Roche	10103586001
Hyaluronidase	Sigma	H4272
RPMI	Gibco	21875-034
DMEM	Gibco	41965-039
DMEM/F12	Sigma	D6421
FBS	Gibco	12657
Pen/Strep	Biological Industries	03-031-1B
HEPES	Sigma	H0887
Glutamine	Biological Industries	03-020-1B
Fungizone (Amphotericin B)	Biological Industries	03-028-1B
Gentamycin	Biological Industries	03-035-1B
MCP-1 ELISA	Peprotech	900-k126
CCL2 ELISA	Peprotech	900-m31
Human Cytokine Array G5	RayBiotech	#AAH-Cyt-G5
shPYK2	Sigma	TRCN00000231519
Protein Assay Reagent (Bradford)	BioRad	5000006
ECL Substrate	BioRad	1705060
PMA	Sigma	P1585
Trypsin	Sigma	T1426
MG132	Calbiochem	474790
LY411575	Sigma	SML0649
JetPRIME	PolyPlus Transfection	114-01
TRI Reagent	Sigma	93289
High-Capacity cDNA Reverse Transcription Kit	Applied Biosystems	4368814
SYBR Green I	Roche	04707516001
BD Cytofix/Cytoperm Kit	BD Bioscience	545714
Streptavidin MicroBeads	Miltenyi Biotec	130-048-101
LS MACS columns	Miltenyi Biotec	130-042-401
True Nuclear Transcription Factor Buffer Set	Biolegend	425401

Table S3. Genotyping primers

Gene	Forward (5’ – 3’)	Reverse (5’ – 3’)
Cre	GCATTACCGGTCGATGCAACGAGTG	GAACGCTAGAGCCTGTTTTGCACGTTC
Cx3cr1	GGTTCTTGCGAACCTCATCAC	GCAGGACAAGCATAGCAGTC
Pyk2 floxed	GAGAGTGCTGGGTACTCCAGACTCAGATAG	TTCAGGAACACCAGAGAACTAGGGTGG
Pyk2 KO	TGTGCTCAGAGAAAAACGGAGGAACCCT	CATTGATTCCTGCTTCAGCCCTGGTCTAA and GCCCATCGGGGCGATTTAAATATAATTCG

Table S4. qRT-PCR primers

Target	Primer F	Primer R
Rps29	GGTCACCAGCAGCTCTACTG	GTCAACTTAATGAAGCCTATGT
ACTB	CATGAAGATCAAGATCATCGCC	ACATCTGCTGGAAGGTGGACA
Ptk2b	CGGCAAGTACAGGTAAGTCGG	AAACTATATGGCAGCCACAGC
CCL2	CAGCCAGATGCAATCAATGCC	TGGAATCCTGAACCCACTTCT
Ccl2	TAAAAACCTGGATCGGAACCAAA	GCATTAGCTTCAGATTTACGGGT
Notch1	GATGGCCTCAATGGGTACAAG	TCGTTGTTGTTGATGTCACAGT
Hes1	CCAGCCAGTGTCAACACGA	AATGCCGGGAGCTATCTTTCT
Ccl22	TCTTGCTGTGGCAATTCAGA	GAGGGTGACGGATGTAGTCC
Ccl17	TACCATGAGGTCACTTCAGATGC	GCACTCTCGGCCTACATTGG
Ym-1	ACCCCGTGCCTGTGTACTCACCT	CACTGAACGGGGCAGGTCCAAA
Mrc1	TTGGACGGATAGATGGAGGG	CCAGGCAGTTGAGGAGGTTC
Cd163	TGTGCAGTAACGGCTGGAG	ATCATGTTTGCAGTCCCAAAGA
Arg1	CCACAGTCTGGCAGTTGGAAG	GGTTGTCAGGGGAGTGTTGATG
Fizz-1	TCCAGCTGATGGTCCCAGTG	GAGGCCCATCTGTTCATAGTCTT
Il1b	ACGGACCCCAAAAGATGAAG	TACTGCCTGCCTGAAGCTCT
Il6	GGGAAATCGTGGAAATGAGA	CCAGTTTGGTAGCATCCATCA
Vegfa	GCACATAGAGAGAATGAGCTTCC	CTCCGCTCTGAACAAGGCT
Ang2	CCTCGACTACGACGACTCAGT	TCTGCACCACATTCTGTTGGA
Tymp	CGCGGTGATAGATGGAAGAGC	CACACCTCCTGTGGAGTGTT
Plau	GCGCCTTGGTGGTGAAAAAC	TTGTAGGACACGCATACACCT
Pdl2	CATCGCTTTGATCTTCCTGG	CCTGAAAGTCATTAGGAGCC
Stab1	GGCAGACGGTACGGTCTAAAC	AGCGGCAGTCCAGAAGTATCT
Fizz-1	TCCAGCTGATGGTCCCAGTG	GAGGCCCATCTGTTCATAGTCTT
Csf1r	TGTCATCGAGCCTAGTGGC	GGTCCAAGGTCCAGTAGGG
Il10	CCCAAGTAACCCTTAAAGTCCTGC	ATGCTGCCTGCTCTTACTGACTG
Ccl8	TCTACGCAGTGCTTCTTTGCC	AAGGGGGATCCCTAGCTTTAGTA
Ccl20	GCCTCTCGTACATACAGACGC	CCAGTTCTGCTTTGGATCAGC
Mmp9	GGACCCGAAGCGGACATTG	CGTCGTCGAAATGGGCATCT
Irf4	TCCGACAGTGGTTGATCGAC	CCTCACGATTGTAGTCCTGCTT

Table S5: Antibodies

Target	Company	Catalogue #	Method
Human PYK2	Litvak et al 2000		WB / IF
Mouse PYK2	Abcam	ab32571	WB / IHC
FAK	Santa Cruz	932	WB
pFAK (Y397)	Cell Signaling	8556	WB
F4/80	Invitrogen	14-4801-82	IHC / IF
CD206	R&D	AF2535	IF
CD68	Santa Cruz	20060	IF
α-Tubulin	Sigma	T8203	WB
β-Actin	GenScript	A00702	WB
p-STAT6 (Y641)	Cell Signaling	56554	WB / IF
STAT6	Santa Cruz	1689	WB / IF
IL4R	R&D	FAB530F	WB
IL4R	Santa Cruz	165974	IF
CCR2	Biolegend	150607	WB
CXCR4	Biolegend	306501	WB
CD31	Abcam	124432	IF
NOTCH1 ICD	Cell Signaling	4147	WB
NOTCH1 Full lenght	Cell Signaling	3608	WB
Biotin-anti goat	Jackson	705-065-147	IF
Streptavidin-Cy3	Jackson	016-160-084	IF
Alexa-F488 anti-mouse	ThermoFisher	A28175	IF
Cy3-anti rabbit	Jackson	711-165-152	IF
Alexa-F488 anti-rabbit	Jackson	111-545-003	IF
Cy3-anti goat	Jackson	705-165-147	IF
Cy2-anti goat	Jackson	705-225-147	IF
Cy3-anti rat	Jackson	712-165-153	IF
HRP-anti rabbit	Cell Signaling	7074	WB
HRP-anti mouse	Jackson	115-035-003	WB
HRP-anti rat	Jackson	112-035-003	WB
HRP-anti goat	Jackson	705-035-003	WB
CD115- Biotin (AFS98)	Biolegend	135507	MACS
αSMA	Sigma	A2547	IF
CD3	BioRad	MCA1477	IF
Ly6C (ER-MP20)	Novus Biologicals	NB100-65413	IF
NK1.1	CST	39197	IF
CK8 (TROMA-8)	Millipore	MABT329	IF
CD45	Biorad	MCA1031G	IF

WB Western blotting, IF Immunofluorescence, IHC Immunohistochemistry

Table S6: FACS antibodies

Target	Fluorophore	Clone	Company	Catalogue #
TrueStain FcX™			Biolegend	156603
LIVE/DEAD Fixable Blue			ThermoFisher	L23105
CD45	APC-Cy7	30-F11	Biolegend	103115

CD11b	FITC	M1/70	Biolegend	101205
F4/80	PE	bm8	Biolegend	123109
Ly6C	BV605	HK1.4	Biolegend	121035
Ly6G	AlexaFluor 700	1A8	Biolegend	127621
SiglecF	APC	S1707L	Biolegend	155507
CD45	AF700	30-F11	Biolegend	103127
CD11b	FITC	M1/70	Biolegend	101205
CD11b	BV605	M1/70	Biolegend	101237
CD11b	PerCP-Cy5.5	M1/70	Biolegend	101227
F4/80	BV785	BM8	Biolegend	123141
F4/80	PE-Cy7	BM8	Biolegend	123111
Ly6C	APC-Cy7	HK1.4	Biolegend	121025
Ly6G	PerCP	1A8	Biolegend	127653
Siglec F	Biotin	S1707L	Biolegend	155512
Streptavidin	PE-Cy7		Biolegend	405206
Siglec F	FITC	S1707L	Biolegend	155503
MhcII	Pacific Blue	AF6-120.1	Biolegend	116421
CXCR4	APC	L276F12	Biolegend	146507
CCR2	PE	475301	R&D	FAB5538T-025
CD124	PE	I015F8	Biolegend	144803

Figure S1

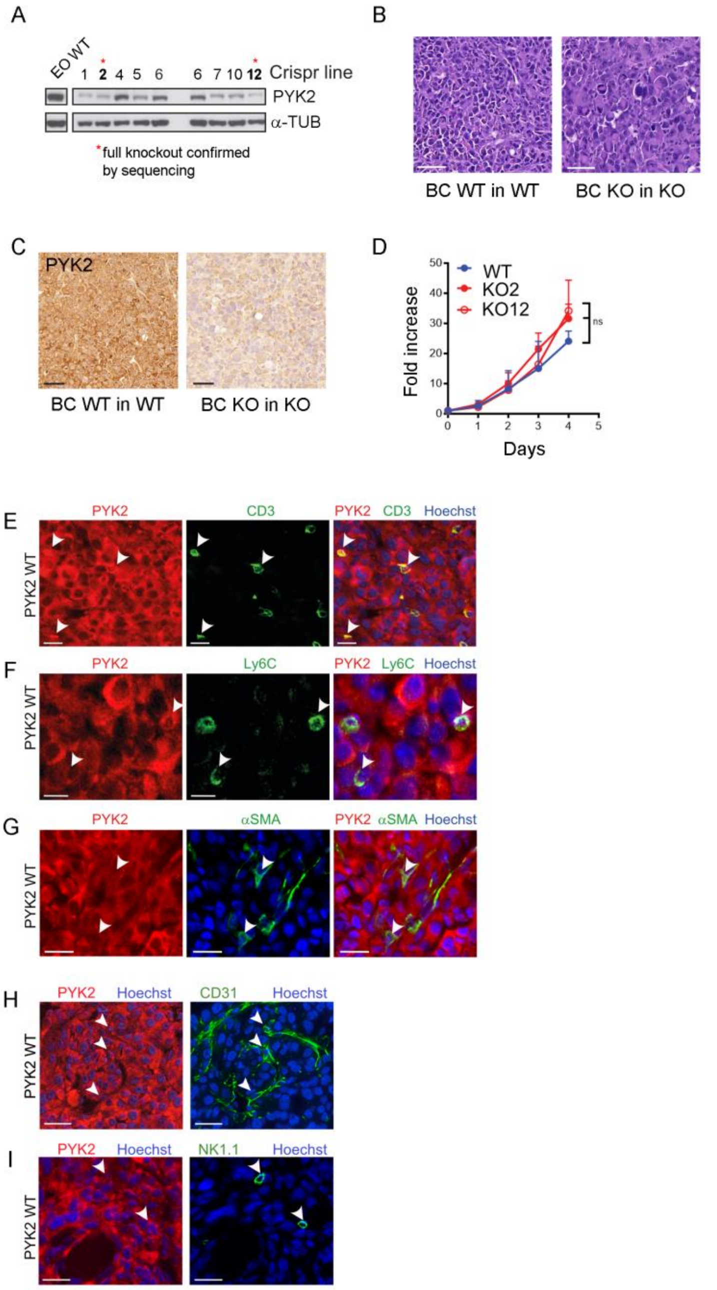

A
EO WT
1 2 4 5 6 6 7 10 12 Crispr line
PYK2
α-TUB
*full knockout confirmed by sequencing
B
BC WT in WT
BC KO in KO
C
PYK2
BC WT in WT
BC KO in KO
D
WT
KO2
KO12
ns
Fold increase
Days
E
PYK2
CD3
PYK2 CD3 Hoechst
PYK2 WT
F
PYK2
Ly6C
PYK2 Ly6C Hoechst
PYK2 WT
G
PYK2
αSMA
PYK2 αSMA Hoechst
PYK2 WT
H
PYK2 Hoechst
CD31 Hoechst
PYK2 WT
I
PYK2 Hoechst
NK1.1 Hoechst
PYK2 WT

Figure S2

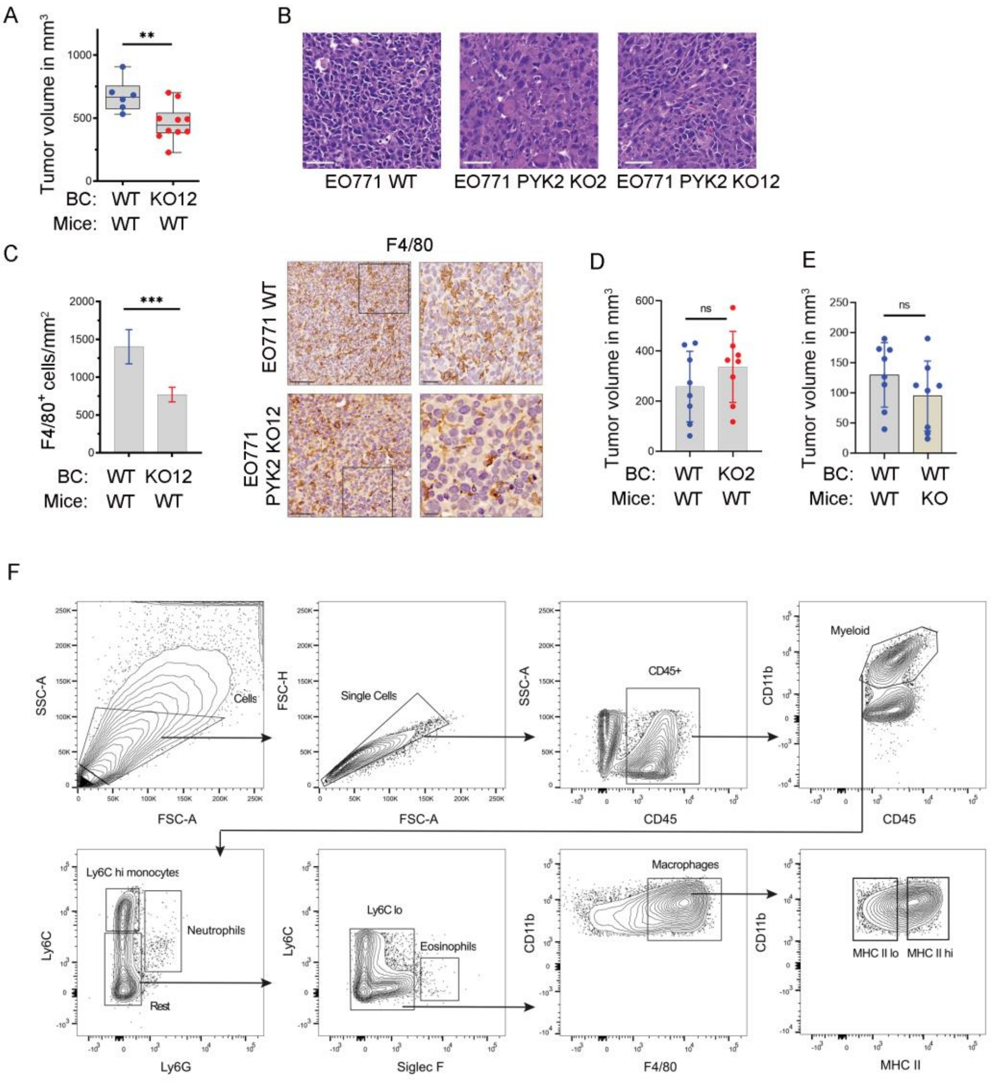

A
Tumor volume in mm3
**
BC: WT KO12
Mice: WT WT
B
EO771 WT
EO771 PYK2 KO2
EO771 PYK2 KO12
C
F4/80+ cells/mm2
***
BC: WT KO12
Mice: WT WT
F4/80
EO771 WT
EO771 PYK2 KO12
D
Tumor volume in mm3
ns
BC: WT KO2
Mice: WT WT
E
Tumor volume in mm3
ns
BC: WT WT
Mice: WT KO
F
SSC-A
FSC-A
Cells
FSC-H
Single Cells
CD45+
CD45
CD11b
Myeloid
Ly6C
Ly6G
Ly6C hi monocytes
Neutrophils
Rest
Siglec F
Ly6C lo
Eosinophils
F4/80
Macrophages
MHC II
MHC II lo
MHC II hi

Figure S3

A

Rel. Signal in shPyk2	Cytokine Target
0,042	IL-1b
0,212	IL-4
0,360	IL-6
0,369	GM-CSF
0,423	IGF-I
0,599	Osteopontin
0,606	MCSF
0,654	ENA-78

Blue: immunosuppressive

Red: angiogenesis-promoting

Green: myeloid cell-attracting

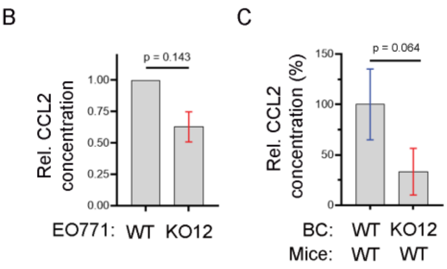

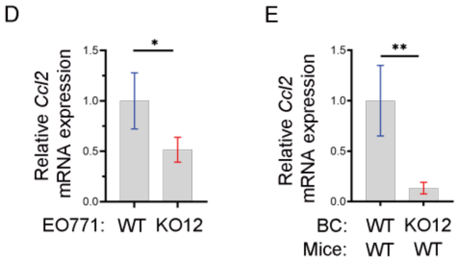

Figure S4

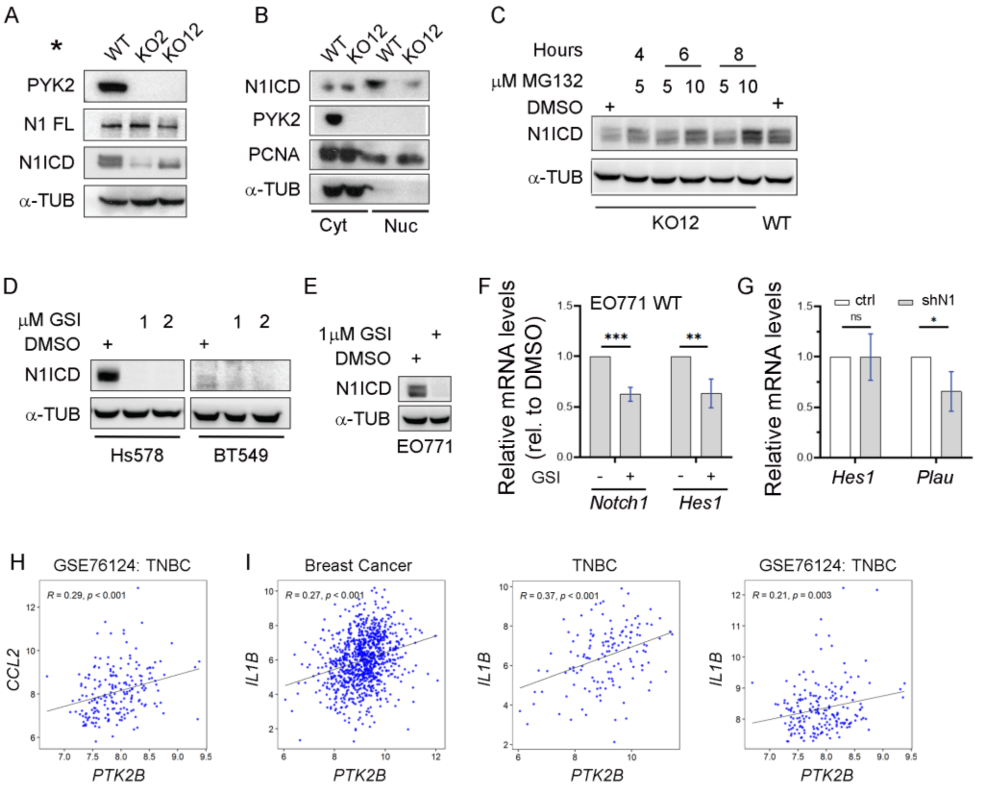
A
*
WT
KO2
KO12
PYK2
N1 FL
N1ICD
α-TUB
B
WT
KO12
WT
KO12
N1ICD
PYK2
PCNA
α-TUB
Cyt
Nuc
C
Hours
4
6
8
μM MG132
5 5 10 5 10
DMSO
+
+
N1ICD
α-TUB
KO12
WT
D
μM GSI
1 2
1 2
DMSO
+
+
N1ICD
α-TUB
Hs578
BT549
E
1μM GSI
+
DMSO
+
N1ICD
α-TUB
EO771
F
EO771 WT
Relative mRNA levels (rel. to DMSO)
***
**
GSI
- + - +
Notch1
Hes1
G
ctrl
shN1
Relative mRNA levels
ns
*
Hes1
Plau
H
GSE76124: TNBC
R = 0.29, p < 0.001
CCL2
PTK2B
I
Breast Cancer
R = 0.27, p < 0.001
IL1B
PTK2B
TNBC
R = 0.37, p < 0.001
IL1B
PTK2B
GSE76124: TNBC
R = 0.21, p = 0.003
IL1B
PTK2B

Figure S5

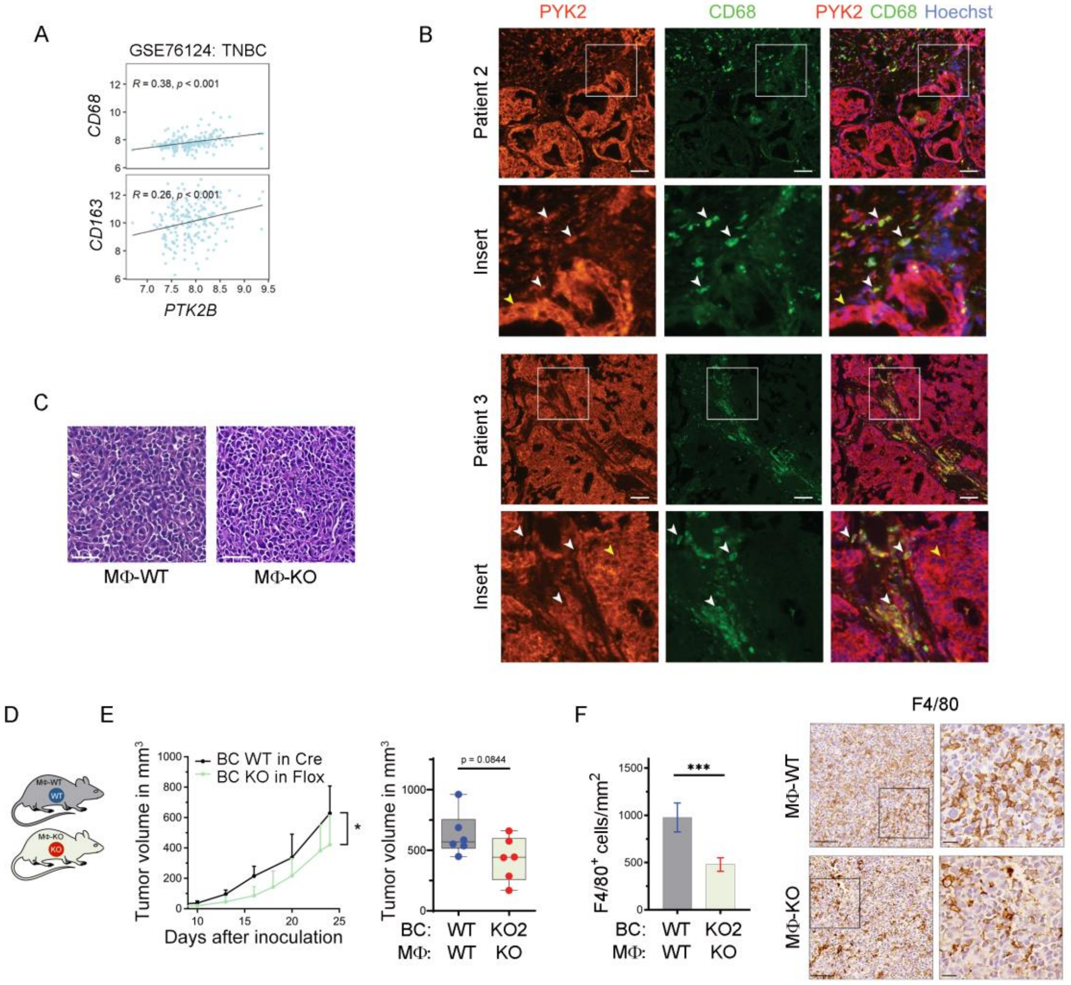
A
GSE76124: TNBC
R = 0.38, p < 0.001
CD68
R = 0.26, p < 0.001
CD163
PTK2B
B
PYK2
CD68
PYK2 CD68 Hoechst
Patient 2
Insert
Patient 3
Insert
C
MΦ-WT
MΦ-KO
D
MΦ-WT
MΦ-KO
E
BC WT in Cre
BC KO in Flox
Tumor volume in mm3
Days after inoculation
p = 0.0844
BC: WT KO2
MΦ: WT KO
F
F4/80
F4/80+ cells/mm2
***
BC: WT KO2
MΦ: WT KO
MΦ-WT
MΦ-KO

Figure S6

A

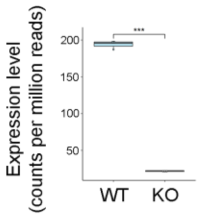
Expression level
(counts per million reads)
200
150
100
50
***
WT
KO

B

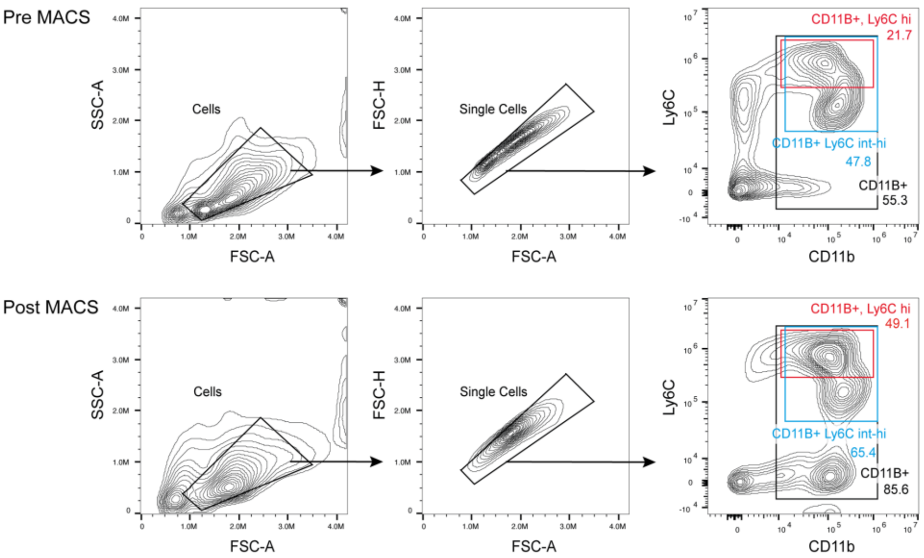
Pre MACS
SSC-A
FSC-A
Cells
FSC-H
Single Cells
Ly6C
CD11b
CD11B+, Ly6C hi
21.7
CD11B+ Ly6C int-hi
47.8
CD11B+
55.3
Post MACS
Cells
Single Cells
CD11B+, Ly6C hi
49.1
CD11B+ Ly6C int-hi
65.4
CD11B+
85.6

Figure S7

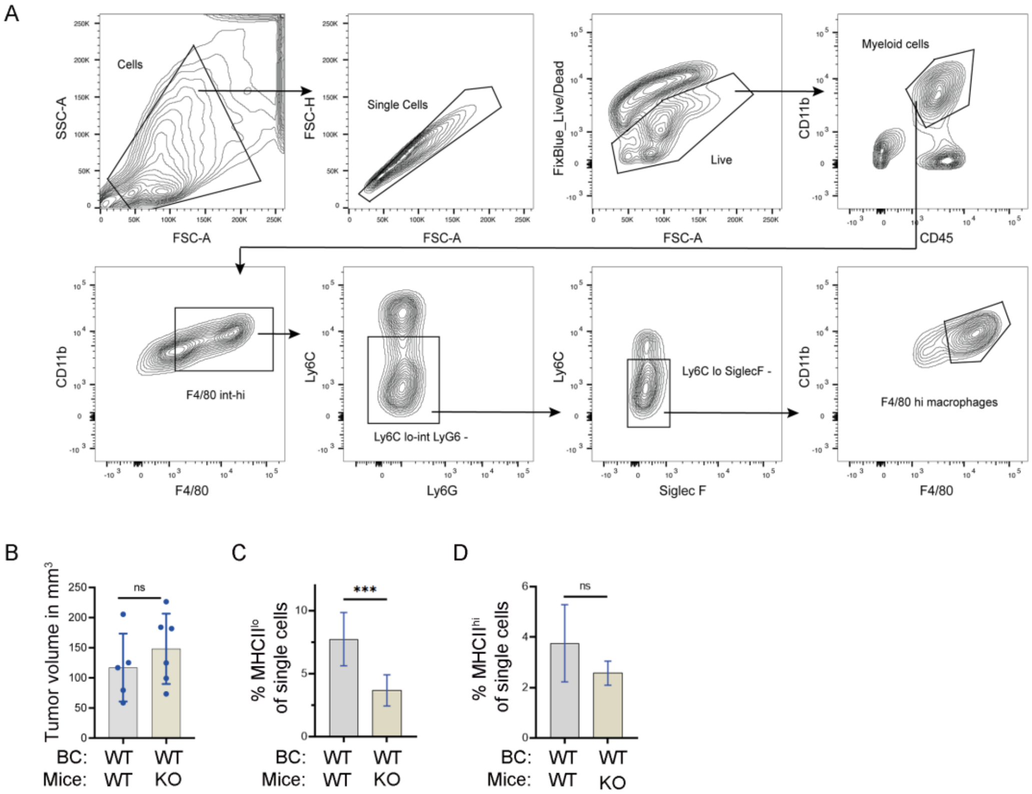
A
Cells
SSC-A
FSC-A
Single Cells
FSC-H
FixBlue_Live/Dead
Live
Myeloid cells
CD11b
CD45
F4/80 int-hi
F4/80
Ly6C
Ly6C lo-int LyG6 -
Ly6G
Ly6C lo SiglecF -
Siglec F
F4/80 hi macrophages
B
Tumor volume in mm3
ns
BC: WT WT
Mice: WT KO
C
% MHCIIlo of single cells
***
BC: WT WT
Mice: WT KO
D
% MHCIIhi of single cells
ns
BC: WT WT
Mice: WT KO
